# Impacts of COVID-19 lockdowns and stimulus payments on low-income population’s spending in the United States

**DOI:** 10.1371/journal.pone.0256407

**Published:** 2021-09-08

**Authors:** Kangli Li, Natasha Zhang Foutz, Yuxin Cai, Yunlei Liang, Song Gao

**Affiliations:** 1 Department of Agricultural and Applied Economics, University of Wisconsin-Madison, Madison, Wisconsin, United States of America; 2 McIntire School of Commerce, University of Virginia, Charlottesville, Virginia, United States of America; 3 Geospatial Data Science Lab, Department of Geography, University of Wisconsin-Madison, Madison, Wisconsin, United States of America; GGS, UNITED STATES

## Abstract

The COVID-19 pandemic has profoundly impacted the economy and human lives worldwide, particularly the vulnerable low-income population. We employ a large panel data of 5.6 million daily transactions from 2.6 million debit cards owned by the low-income population in the U.S. to quantify the joint impacts of the state lockdowns and stimulus payments on this population’s spending along the inter-temporal, geo-spatial, and cross-categorical dimensions. Leveraging the difference-in-differences analyses at the per card and zip code levels, we uncover three key findings. (1) Inter-temporally, the state lockdowns diminished the daily average spending relative to the same period in 2019 by $3.9 per card and $2,214 per zip code, whereas the stimulus payments elevated the daily average spending by $15.7 per card and $3,307 per zip code. (2) Spatial heterogeneity prevailed: Democratic zip codes displayed much more volatile dynamics, with an initial decline three times that of Republican zip codes, followed by a higher rebound and a net gain after the stimulus payments; also, Southwest exhibited the highest initial decline whereas Southeast had the largest net gain after the stimulus payments. (3) Across 26 categories, the stimulus payments promoted spending in those categories that enhanced public health and charitable donations, reduced food insecurity and digital divide, while having also stimulated non-essential and even undesirable categories, such as liquor and cigar. In addition, spatial association analysis was employed to identify spatial dependency and local hot spots of spending changes at the county level. Overall, these analyses reveal the imperative need for more geo- and category-targeted stimulus programs, as well as more effective and strategic policy communications, to protect and promote the well-being of the low-income population during public health and economic crises.

## Introduction

The novel coronavirus disease 2019 (COVID-19) pandemic, one of the most devastating public health crises in the modern human history, has profoundly impacted the economy and human lives [1]. The U.S. real gross domestic product (GDP) decreased 5% during the first quarter of 2020 and 31.7% during the second quarter (according to the Bureau of Economic Analysis [2]); and the unemployment rate reached 8.4% in August (according to the U.S. Bureau of Labor Statistics [3]). While the latest research has focused on the impact of human mobility restrictions on the virus spread, disparities in COVID-19 transmission, and the environmental and macroeconomic consequences under the COVID-19 lockdowns [4–13], our research focuses on the dynamics of micro-level consumer spending during the pandemic that is of vital importance to the economic recovery. Our research also enriches the latest public health research by examining the shift of consumer spending critical to financial health, in addition to physical and mental health [14].

Most importantly, although the pandemic has greatly impacted the entire U.S. population’s income, wealth, and spending [15], the low-income population, defined in our context as having an annual personal income below the 2019 U.S. real median of $35,977 (https://www.census.gov/topics/income-poverty/income.html), was hit the hardest by job and income loss during the pandemic, especially after the state lockdowns starting from California on March 19, 2020 [16]. A recent study shows that low-income communities are less likely or cannot afford to comply with stay-at-home orders [17]. In addition, income, along with race, has also become a major predictor of COVID-19 infections [18–20]. Furthermore, to mitigate the economic fallout and aid the low-income population, $300 billion one-time stimulus payments were distributed starting from April 11, 2020, as part of a $2 trillion economic stimulus bill (CARES Act), the largest economic stimulus package in the U.S. history [21]. Eligible individuals were given $1,200 per person (with adjusted annual gross income < $75,000) or $2,400 per married couple (< $150,000), and $500 per child.

All the above indicates that the COVID-19 pandemic and the mitigation policies have disproportionately impacted the most vulnerable low-income population in the U.S. [17, 20, 22–24]. Our research thus examines this population, in a different social context than what has been historically studied, such as social inequality and political polarization [25]. Our focus on this vulnerable yet under-studied low-income population also differentiates from the recent studies that either under-represent or exclude this population [26, 27]. Our research is also grounded on a rich consumer behavior literature that examines consumer well-being and spending behavior under financial or emotional constraints. Under such constraints, consumers become more concerned about lasting utilities of their purchases, hence preferring material goods over experiences [28]. Resource scarcity will guide consumers towards advancing their own welfare [29]. Budget allocations across categories will also change during economic downturns [30]. Research on mortality salience, i.e., reminder of own impending mortality, further suggests that exposure to death-related stimuli will increase purchasing and consumption, especially among low-self-esteem consumers [31]. Exposure to death-related information about others in the media will also shift consumers’ focus from extrinsic to intrinsic values [32]. Moreover, behavioral theories on mental accounting state that people track their expenditures using cognitive categories or “mental accounts”. An example in our context is that consumers put the stimulus payments received into a separate mental account from their other sources of income. Once a mental account is established, purchases highly congruent with the purpose of the mental account will be more preferred [33]. These behavioral theories have foretold some spending patterns that we will discover from our data, such as the initial decrease in spending, likely due to stress or resource constraints, followed by the subsequent increase in spending upon the receipt of the stimulus payments, potentially arising from mental accounting, as well as the heterogeneity in the spending shifts across categories as a result of the cross-category budget re-allocation.

Specifically, our research aims to address the following key research questions (RQs):

RQ1: What are the inter-temporal impacts of the state lockdowns and stimulus payments on the low-income population’s spending during the COVID-19 pandemic?
RQ2: How do these impacts vary across major economic regions, neighbouring locations, and areas of different political affiliations?
RQ3: Do some categories exhibit desirable or undesirable shifts in spending?
RQ4: Can we design better policies to protect and promote the well-being of the low-income population during public health and economic crises?

To address the above research questions of interest, we leverage the state lockdowns starting from March 19, 2020 and distributions of the stimulus payments starting from April 11, 2020 as two major natural shocks in a Difference-in-Differences (DID) analysis framework. Analyzing a national panel data set of more than 5.6 million daily transactions from over 2.6 million consumer debit cards owned by the low-income consumers in the U.S., we compare the daily spending difference of the same period from 2019 to 2020, before and after the initial lockdown (and each state’s lockdown as a robustness check), and before and after the stimulus payments. We continue to leverage the geo-spatial analysis to explore the potential heterogeneity of these effects across eight major economic regions, all bordering counties, and Republican versus Democratic leaning zip codes. Finally, we employ a cross-category analysis on 26 categories from 10 major spending groups. Revolving around the above key research questions, we find that (RQ1) inter-temporally, the lockdowns diminished the daily average spending relative to the same period in 2019 by $3.9 per card and $2,214 per zip code, whereas the stimulus payments elevated the daily average spending by $15.7 per card and $3,307 per zip code; (RQ2) spatial heterogeneity prevailed, for instance, Democratic zip codes displayed much more volatile dynamics than Republican ones; (RQ3) across 26 categories, the stimulus payments promoted spending in those essential to the population’s well-being, yet also increased spending in undesirable categories such as liquor and cigar; (RQ4) the discovered geo- and category-heterogeneities call for more geo- and category-targeted stimulus programs to protect the low-income population during the public health and economic crises.

### Contributions

Addressing these important questions will generate crucial insights on human behavior in response to crises, unveil the state of well-being of one of the most vulnerable populations, assess the impact of government mitigation measures, and shed valuable lights on the pressing issues of broad interest, such as social disparity, digital divide, and economic recovery. This research further contributes distinctive inter-temporal, geo-spatial, and cross-categorical angles. Inter-temporally, in contrast to the present studies that have examined the impacts of either the lockdowns alone [27, 34, 35] or stimulus payments alone [36, 37], our research examines the joint impacts of the lockdowns and low-income focused stimulus payments, thus offering a more complete portrayal of the decline-then-rebound dynamics of the low-income population’s spending. Geo-spatially, our analyses at the region, county, and zip code levels reveal great geographical heterogeneity in the spending shifts, strong correlations across bordering counties, and sharp contrasts between Republican and Democratic zip codes, all important and novel additions to the literature. Cross-categorically, compared to the studies that examine either aggregate spending or spending over a small number of categories [27, 34, 36, 38], our research offers highly granular analyses spanning 26 categories across 10 major spending groups, thus delineating to our knowledge the most comprehensive picture of the low-income population’s spending behavior during this unprecedented crisis. From a policy perspective, this research uncovers the need for more geo- and category-targeted stimulus programs in light of the strong geo- and cross-category heterogeneities in the spending dynamics, thus enriching the literature that has focused on Marginal Propensity to Consume (MPC)-oriented policy recommendations [36, 37].

The remainder of the paper is organized as follows. We will first introduce the data sets used in this study. Then, the DID statistical analysis and the spatial association analysis methods are presented, followed by the analysis results revolving around each research question. Finally, we will discuss the broader implications of this study and draw conclusions.

## Data

**Fig 1:**
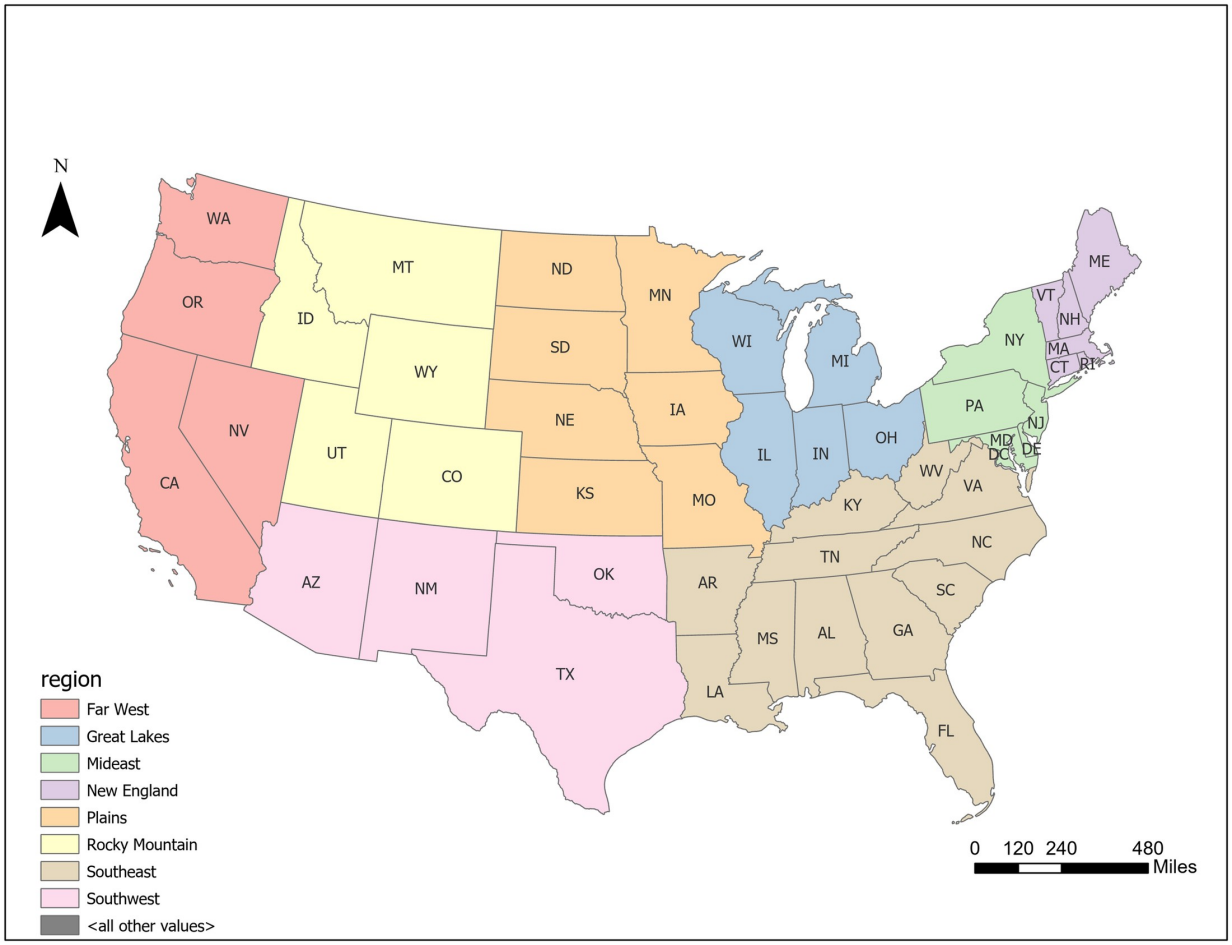
The eight U.S. economic regions defined by the Department of Commerce Bureau of Economic Analysis: New England, Mideast, Southeast, Great Lakes, Plains, Rocky Mountains, Southwest, and Far West.

We leverage the U.S. Census data and Facteus financial transaction data of a representative sample of the low-income population sourced from over 1,000 financial institutions throughout the U.S. The data cover more than 5.6 million daily transactions from 2.6 million debit cards owned by a panel of low-income consumers with an average personal income of $22,000 residing in 21,855 zip codes from January 1, 2019 to May 3, 2020. These debit cards encompass four types: debit cards issued by non-traditional mobile banks (i.e., challenger banks), general-purpose cards by general-purpose stores such as Walmart, payroll cards by employers, and government cards. These consumers use the cards as their primary bank accounts for deposits and spending. While including both online and offline transactions, the data do not distinguish them. Nor do the data provide details on individual transactions or additional demographic characteristics of the cardholders. We examine the daily spending behavior spanning a wide spectrum of categories at both the per-card and zip code levels. The spending is further classified into 26 categories from 10 major spending groups based on the Visa Merchant Category Codes (MCC)—a global standard to identify the merchant of each transaction (S1 Table in S1 File). In addition, we leverage the following U.S. Census data: (1) political affiliation, where a zip code is labeled as Republican (Democratic) if its corresponding county voted (did not vote) for Trump in the 2016 presidential election [39]; (2) eight economic regions as defined by the U.S. Department of Commerce Bureau of Economic Analysis [40]: New England, Mideast, Southeast, Great Lakes, Plains, Rocky Mountains, Southwest, and Far West (Fig 1). In addition, we obtain the starting dates of the state lockdowns (i.e., stay-at-home orders) from the New York Times [41].

**Fig 2:**
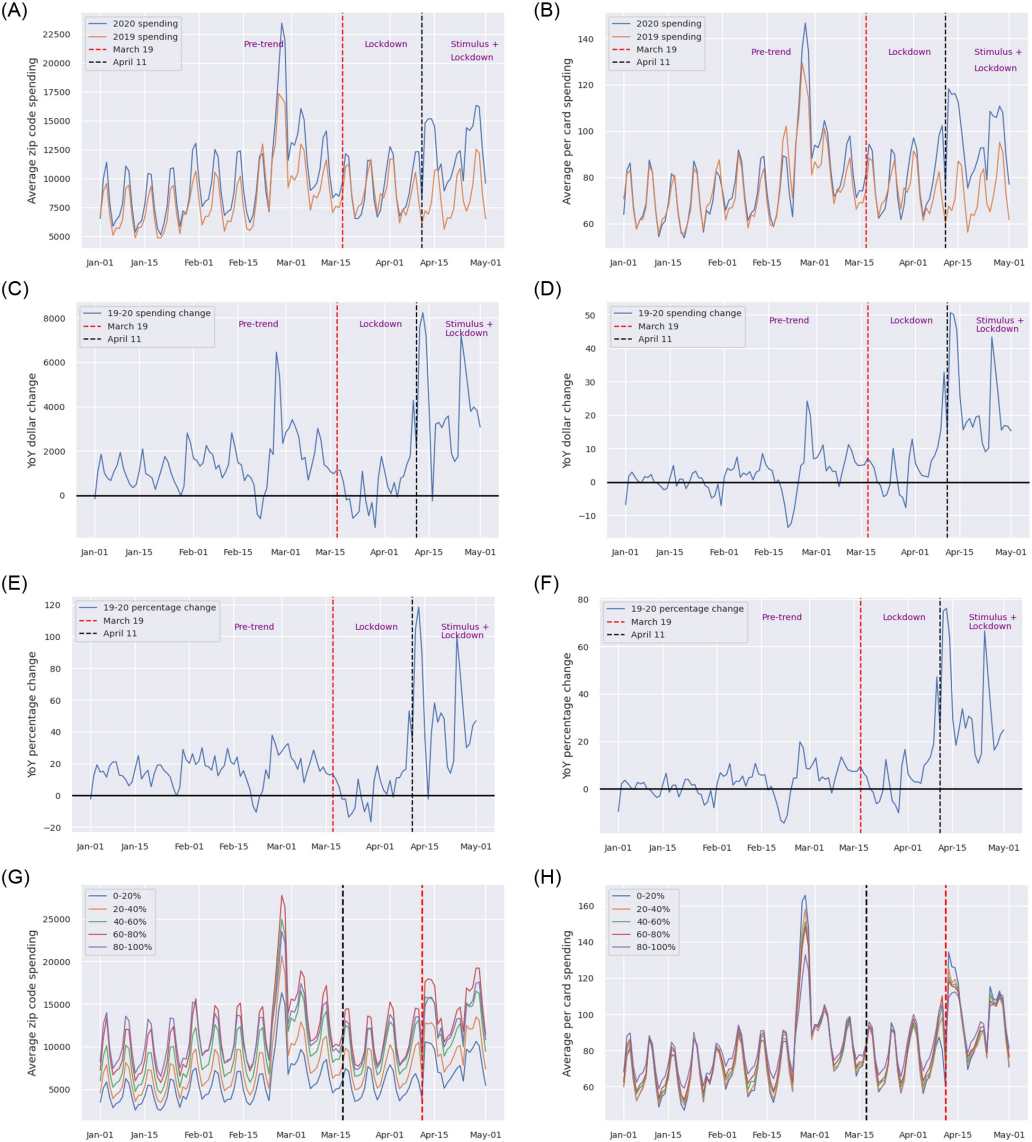
Trends of daily average zip code spending and daily average per card spending from Jan 1 to May 3 of 2019 and 2020. The dates are adjusted to match the day of the same week across 2019 and 2020. (a) Trend of daily average zip code spending. (b) Trend of daily average per card spending. (c) Trend of YoY dollar change in daily average zip code spending. (d) Trend of YoY dollar change in daily average per card spending. (e) Trend of YoY percentage change in daily average zip code spending. (f) Trend of YoY percentage change in daily average per card spending. (g) Trend of daily average zip code spending by income quintile. (h) Trend of daily average per card spending by income quintile.

Fig 2a depicts the inter-temporal trends of the daily average spending at the zip code level in 2019 and 2020. A zip code in our sample sees on average $5,000-$20,000 daily expenditure. The trends follow a noticeable weekly cycle, with more spending on Thursday and Friday and less on other days in most weeks. The spikes in late February across both years largely arise from the federal earned income tax credit (EITC), a big financial boost to this population. We hence use the 2019 spending to control for these confounding inter-temporal factors, such as the weekly cycle, February spikes, and seasonality. Specifically, we subtract the daily average spending at the zip code level by that of the same day in 2019 (with minor adjustment to match the day-of-the-week), producing the year-over-year (YoY) dollar change (dollar change hereafter). Fig 2c plots the temporal trend of the dollar change, showing that a zip code in 2020 typically exhibits a $2,000 increase in spending by the same panel of consumers, with an enlarged gap in late February due to the tax credit. The trend hit below the zero line following the initial lockdown, and then sharply increased to $8,000 after the stimulus payments. Having teased out the confounding time factors, we can now estimate the effect of the lockdown by subtracting the dollar change after the initial lockdown by that before; similarly for the case of the stimulus payments. Fig 2e presents the temporal trend of the corresponding YoY percentage change (percentage change hereafter). Compared to the dollar change in Fig 2c, the percentage change is much more stable: the increase in late February is not significantly different from that in January and early February. In the following sections, we will use both the dollar change and percentage change as our dependent variables and discuss the results from both. Nonetheless, we will focus on the percentage change when comparing across categories. Fig 2b, 2d and 2f show similar trends in the daily average per card spending per zip code, and corresponding dollar change and percentage change over the same period, respectively. Fig 2g and 2h present the trends of the daily average zip code and per card spending, respectively, by the residing county’s Census income quintile. All zip codes exhibit a similar temporal pattern in the daily average per card spending (Fig 2h), confirming that the data cover similar kinds of low-income consumers regardless of whether they reside in higher- or lower-income areas.

## Methods

### Difference-in-Differences (DID) statistical analysis

We implement a difference-in-differences (DID) model to estimate the effects of the lockdowns and stimulus payments on spending. As described earlier, the dependent variable is either the dollar change or percentage change. The model then captures the shift of the dependent variable before versus after the initial lockdown on March 19, 2020 (first treatment), and before versus after the distribution of the stimulus payments on April 11, 2020 (second treatment). Formally, the empirical specification for the dollar change goes as follows.

$$\begin{array}{c} \Delta Y_{zd}=\beta *D1+\gamma *D2+\theta_{zm}+\epsilon_{zd}, \end{array}$$ (1)

Where Δ*Y*_*zd*_ is the dollar change for zip code *z* on a day *d*, that is, Δ*Y*_*zd*_ = *Y*_*zd*,2020_ − *Y*_*zd*,2019_. We have also estimated the same model for the percentage change, in which case $\Delta Y_{zd}=\frac{Y_{zd,2020}-Y_{zd,2019}}{Y_{zd,2019}}*100\%$. The indicator function D1 = 1{d≥03-19} equals 1 if a date *d* is on or after March 19, 2020, the date of initial lockdown by California; or 0 if otherwise. Therefore, its coefficient *β* captures the effect of the initial lockdown on spending relative to the pre-lockdown period in 2020, while controlling for same-day spending in 2019. Similarly, the indicator function D2 = 1{d≥04-11} equals 1 if a date *d* is on or after the distribution of the stimulus payments on April 11, 2020; or 0 if otherwise. Hence, its coefficient *γ* captures the *net* impact of the stimulus payments on household spending; and (*β* + *γ*) together delineates the joint effect of both the lockdown and stimulus payments after April 11, 2020. Essentially, this approach uses a zip code’s past self in 2019 as the control group for its spending in 2020. The DID method involves two differences: the first is to subtract the daily spending in 2020 by the same-day spending in 2019 to tease out the inter-temporal confounders; and the second difference is to compare the YoY change in spending before and after each treatment (the lockdown or stimulus payments).

We also add a zip code-month fixed effect *θ*_*zm*_ to further control for the unobserved local (zip code *z*) and temporal (monthly *m*) factors, such as seasonal dynamics, population density, demographics, rural/urban, and other idiosyncratic differences across zip codes (local news, price promotions, and consumption-related shocks). In other words, it controls for the unobserved spatial differences that might cause the spending variations, and further teases out the seasonal changes down to the month level not fully captured by the year-over-year DID. Technically, estimating this fixed effect involves adding a large number of zip code-month specific dummies into the model. By doing so, all the aforementioned differences across zip codes and months are teased out, hence producing clean estimates of the impacts of the lockdowns and stimulus payments on spending of core interest to us. The final error term in the model *ϵ*_*zd*_ measures the unobservable randomness not fully captured by the two dummies of core interest and the fixed effects. Both DID and fixed effects ensure that *ϵ*_*zd*_ merely captures the remaining randomness, hence producing the unbiased estimators *β* and *γ*.

#### Robustness check

We further calibrate a specification with the staggered lockdown dates across different states, as opposed to a single date of the initial lockdown. The key findings remain (S2 Table in S1 File).

### Spatial association analysis

To further analyze the degree of spatial dependency in the low-income population’s spending shifts across geographic regions, we leverage the spatial association (auto-correlation) statistics [42, 43]. Specifically, we use the *Global Moran’s I statistic* [44] to examine if there exists a spatially clustered or dispersed distribution of the spending shifts across the U.S. We also compute the *Local Moran’s I statistic* (i.e., Anselin’s Local Indices of Spatial Association) to identify local hot spots and cold spots [45]. These spatial association statistics require a spatial weighting matrix that reflects the spatial relationship between each location (such as a county in our case) and its neighbours, e.g., the distance-to-neighbour matrix or the binary adjacency matrix in which the element value is 0 or 1, as determined by whether there is a shared boundary between a center location and its neighbors. These spatial association statistics are compared with a null hypothesis of a complete spatial randomness process. A z-score and a p-value on a two-sided test are derived to evaluate the statistical significance of the indices. Note that the U.S. zip codes do not meet the spatial contiguity requirement. Thus we run all the spatial association statistical tests at the county level via ESRI’s ArcGIS software version 10.7 and use the spatial contiguity/neighbouring constraints to define the spatial weighting matrix.

Specifically, the Global Moran’s I Statistic is defined as [46]:

$$\begin{array}{c} I=\frac{\sum_{i=1}^{n}\sum_{j=1}^{n}W_{i,j}\left(X_{i}-\bar{X}\right)\left(X_{j}-\bar{X}\right)}{\frac{\sum_{i=1}^{n}{\left(X_{i}-\bar{X}\right)}^{2}}{n}\sum_{i=1}^{n}\sum_{j=1}^{n}W_{i,j}} \end{array}$$ (2)

where *X*_*i*_ is the daily county (or per card) spending DID metric (not the DID regression analysis at the zip code level described earlier) for county *i*, which involves two differences: the first is to subtract the daily average county (or per card) spending in 2020 by the same-day spending in 2019; and the second is to subtract this year-over-year dollar change after each treatment by that before each treatment (the lockdowns or stimulus payments, respectively); *X*_*j*_ is the same metric for a different county *j*; $\bar{X}$ is the global mean of the corresponding metric across all counties; *W*_*i*,*j*_ is the spatial weight between county *i* and county *j* (by convention, *W*_*i*,*i*_ = 0 and *W*_*i*,*j*_ = 1 only if *i* and *j* are neighbouring counties); *n* is the total number of counties with observed spending values (i.e., 2,966). The *I* statistic values fall between -1 (towards a dispersed pattern) and +1 (towards a clustered pattern).

The Local Moran’s I statistic is defined as [47]:

$$\begin{array}{c} I_{i}=\frac{\left(X_{i}-\bar{X}\right)\sum_{j=1,j\neq i}^{n}W_{i,j}\left(X_{j}-\bar{X}\right)}{\frac{\sum_{j=1,j\neq i}^{n}{\left(X_{j}-\bar{X}\right)}^{2}}{n-1}} \end{array}$$ (3)

where the notations remain identical to those in the global Moran’s I statistic, except that *I*_*i*_ is a local index for each county *i*. Comparing the observed statistic with a complete spatial randomness process, a high positive z-score indicates that the neighbouring counties have a similar pattern in the spending shift captured by the above DID metric (either high values or low values) to that of the focal county and derives a high-high (or low-low) spatial cluster, whereas a low negative z-score indicates that the neighbouring counties have a reversed spending pattern to that of the focal county and derives a high-low (or low-high) spatial cluster.

## Results

We will now discuss the results revolving around the key research questions raised earlier.

### RQ1: Impacts of lockdowns and stimulus payments on spending over time

Table 1 shows the estimation results of the DID model using the zip code level dollar change and percentage change as the dependent variable, respectively. We see that after the initial lockdown on March 19, 2020, each zip code reduced the dollar change by $2,214.308. The stimulus payments distributed since April 11 reversed the course by $3,307.330, thus essentially increasing the dollar change by $1,093.022 (i.e., $3,307.330 -$2,214.308) after April 11. The percentage change revealed a similar pattern, with the lockdown reducing the percentage change by 23.035 percentage points and stimulus payments reversing the decline by another 38.208 percentage points. Table 2 shows the results using the per card dollar change and percentage change as the dependent variables at the zip code level. There was an average reduction of $3.905 (6.592 percentage points) per card after the initial lockdown and a large increase of $15.774 (25.858 percentage points) after the stimulus payments, resulting in a net increase of $11.869 (19.266 percentage points) per card. The results demonstrate the improved effectiveness of the 2020 stimulus payments in stimulating spending compared to the impacts of the 2001 and 2008 stimulus payments [48–50]. For instance, an average household’s spending was found to rise by 10% in the first week and remained at 1.5–3.8% in the first three months after the 2008 stimulus payments [49].

**Table 1:** Effects of initial lockdown and stimulus payments on zip code spending.

	Dollar Change ($)			Percentage Change (%)		
	All	Republican	Democratic	All	Republican	Democratic
β:1{≥03-19}	-2214.308***	-1298.961***	-3779.522***	-23.035***	-20.924***	-26.645***
	(27.910)	(20.785)	(62.869)	(0.198)	(0.266)	(0.280)
γ:1{≥04-11}	3307.330***	2335.112***	4963.615***	38.208***	40.501***	34.302***
	(43.781)	(35.289)	(99.186)	(0.239)	(0.315)	(0.355)
Adjusted R^2^	0.472	0.424	0.489	0.227	0.218	0.245
N	2521355	1589995	931360	2521355	1589995	931360

*** 0.01

** 0.05

* 0.1

Note: The regressions include the zip code-month fixed effect. The standard errors shown in the parenthesis are clustered at the zip code level.

**Table 2:** Effects of initial lockdown and stimulus payments on per card spending.

	Dollar Change ($)			Percentage Change (%)		
	All	Republican	Democratic	All	Republican	Democratic
β:1{≥03-19}	-3.905***	-3.788***	-4.105***	-6.592***	-6.850***	-6.151***
	(0.125)	(0.164)	(0.191)	(0.183)	(0.244)	(0.267)
γ:1{≥04-11}	15.774***	16.228***	15.000***	25.858***	27.288***	23.421***
	(0.129)	(0.168)	(0.198)	(0.210)	(0.280)	(0.305)
Adjusted R^2^	0.145	0.136	0.165	0.155	0.145	0.175
N	2521355	1589995	931360	2521355	1589995	931360

*** 0.01

** 0.05

* 0.1

Note: The regressions include the zip code-month fixed effects. The standard errors shown in the parenthesis are clustered at the zip code level.

#### Robustness check using the staggered state lockdowns

To check the robustness of the results, we also estimate the proposed model using the staggered state-specific lockdown dates (S2 Table in S1 File). The findings remain consistent, showing a large decline after the lockdowns ($1,689.657 or 19.503 percentage points) and then rebound after the stimulus payments ($3,409.338 or 39.386 percentage points). The magnitude of the initial spending decline is smaller, as compared to the result from using the initial lockdown date of March 19 (Table 1), potentially because the residents in those states with later lockdown dates had started reducing spending before their own states enforced lockdowns, as a result of geographic and social inter-dependence across regions [51].

### RQ2: Impacts of lockdowns and stimulus payments on spending across geographic areas

#### Comparing across zip codes of different political affiliations

We are interested in how the geo-spatial distribution of political affiliation impacts the spending shift, in light of the recent studies showing a relationship between political affiliation and risk perception as well as social distancing compliance. For example, a county’s higher share of the republican presidential votes is associated with less perceived risk and less compliance with social distancing during the COVID-19 pandemic [52–54]. Also, Democrats are less likely to respond to a state-level order issued by a Republican governor [55]. Table 1 clearly demonstrates that the effects of the lockdowns and stimulus payments on the dollar change and percentage change diverge across zip codes of different political affiliations. The Democratic zip codes (i.e., with lower vote shares for Trump during the 2016 presidential election) demonstrated a more volatile spending pattern than the Republic zip codes, with a more dramatic decline followed by a stronger rebound. Specifically, these Democratic zip codes reduced the dollar change nearly three times ($3,779.522) as much as their Republican counterparts ($1,298.961) after the initial lockdown. Such a pronounced initial spending decline is consistent with the higher risk perception in the Democratic areas [52–54]. These zip codes also exhibited a rebound ($4,963.615) more than twice that of the Republican zip codes ($2,335.112) after the stimulus payments. As a result, the Democratic zip codes saw a slightly higher net increase ($1,184.093) than the Republican zip codes ($1,036.151) after the stimulus payments. The percentage change is less dramatic in comparison: the Democratic zip codes displayed a decline of 26.645 percentage points after the initial lockdown, compared to 20.924 in the Republican zip codes (Table 1). Their rebound (34.302 percentage points) was actually smaller than that of the Republican zip codes (40.501), resulting in a smaller net increase (7.657 percentage points) than the Republican zip codes (19.577). We also provide the results regarding the partisan differences across spending categories in the S1 File.

#### Comparing across eight economic regions

To further examine the geographic variations, we estimate the DID model for each of the eight economic regions of the U.S. (Tables 3 and 4). Upon the initial lockdown, Southwest ($3,065.323) and Far West ($2,951.148) exhibited the most pronounced reduction in the daily zip code spending, potentially because several early COVID-19 local clusters occurred in California and Washington states [51, 56]. In contrast, Plains ($1,146.347) and New England ($1,229.282) showed the least reduction upon the initial lockdown; and were among those with the least rebound ($2,101.808 and $1,784.659 respectively) after the stimulus payments. Southwest ($4,330.866) and Southeast ($4,122.993) showed the most rebound. Overall, after the initial spending decline and subsequent rebound, Southeast exhibited the largest net increase of $1,779.651 daily per zip code, followed by Great Lakes ($1,369.69). On the other hand, Far West saw a net reduction of $69.761 daily per zip code even after the boost from the stimulus payments. The percentage change revealed a somewhat different picture (Table 4). After the initial lockdown, New England (-26.860 percentage points) and Mideast (-26.690) experienced the greatest percentage change, whereas Plains the least (18.603). After the stimulus payments, Southeast (45.813) and Great Lakes (41.565) saw the strongest rebound, whereas Rocky Mountains (26.995) and Far West (27.074) the least. The net effect is strongest in Southeast (23.74) and smallest in Far West (0.514). We will later further examine these variations across geographic regions by major spending group and by category.

**Table 3:** Effects of initial lockdown and stimulus payments on dollar change across geographic regions.

	Dependent Variable: Dollar Change ($)							
	New England	Mideast	Southeast	Great Lakes	Plains	Rocky Mountains	Southwest	Far West
β:1{≥03-19}	-1229.282***	-2177.546***	-2343.342***	-2008.290***	-1146.347***	-1722.898***	-3065.323***	-2951.148***
	(65.880)	(80.652)	(53.991)	(70.553)	(56.013)	(99.195)	(94.947)	(83.750)
γ:1{≥04-11}	1784.659***	2673.545***	4122.993***	3377.980***	2101.808***	2095.220***	4330.866***	2881.387***
	(105.530)	(108.504)	(93.169)	(127.670)	(100.927)	(119.693)	(123.740)	(98.974)
Adjusted R^2^	0.465	0.469	0.456	0.556	0.464	0.378	0.441	0.382
N	132596	362047	744803	418274	244021	83057	284190	255411

*** 0.01

** 0.05

* 0.1

Note: The regressions include zip-month fixed effects. The standard errors shown in the parenthesis are clustered at the zip code level.

**Table 4:** Effects of initial lockdown and stimulus payments on percentage change across geographic regions.

	Dependent Variable: Percentage Change (%)							
	New England	Mideast	Southeast	Great Lakes	Plains	Rocky Mountains	Southwest	Far West
β:1{≥03-19}	-26.860***	-26.690***	-22.073***	-24.093***	-18.603***	-21.203***	-18.747***	-26.560***
	(0.948)	(0.555)	(0.357)	(0.509)	(0.680)	(1.177)	(0.472)	(0.557)
γ:1{≥04-11}	33.665***	35.391***	45.813***	41.565***	36.239***	26.995***	34.071***	27.074***
	(1.113)	(0.643)	(0.426)	(0.629)	(0.811)	(1.345)	(0.581)	(0.639)
Adjusted R^2^	0.198	0.209	0.233	0.248	0.186	0.195	0.223	0.216
N	132596	362047	744803	418274	244021	83057	284190	255411

*** 0.01

** 0.05

* 0.1

Note: The regressions include zip-month fixed effects. The standard errors shown in the parenthesis are clustered at zip code level.

#### Understanding county level spatial association

We also analyze and visualize the spatial distributions of the county (and per card) spending DID metrics in S1 and S2 Figs in S1 File. We then quantify the spatial association patterns across neighboring counties using the Moran’s I statistic [43, 44]. As shown in S3 Table in S1 File, the global Moran’s I using the county spending DID metric are 0.019 (after the initial lockdown) and 0.115 (after the stimulus payments), respectively; and the global Moran’s I using the per card DID metric are 0.100 (after the lockdown) and 0.252 (after the stimulus payments), respectively. All z-scores are larger than 1.96, and thus the spatial clustered patterns are statistically significant (p-value < 0.05). Recent studies have also identified the spatial association patterns of the COVID-19 spread and human mobility in the U.S. [51, 56, 57]. Our study thus adds to this literature with a better understanding of the spatial association patterns of consumer spending during the COVID-19 pandemic and reveals that spatial dependence may underlie the observed consumer spending patterns.

**Fig 3:**
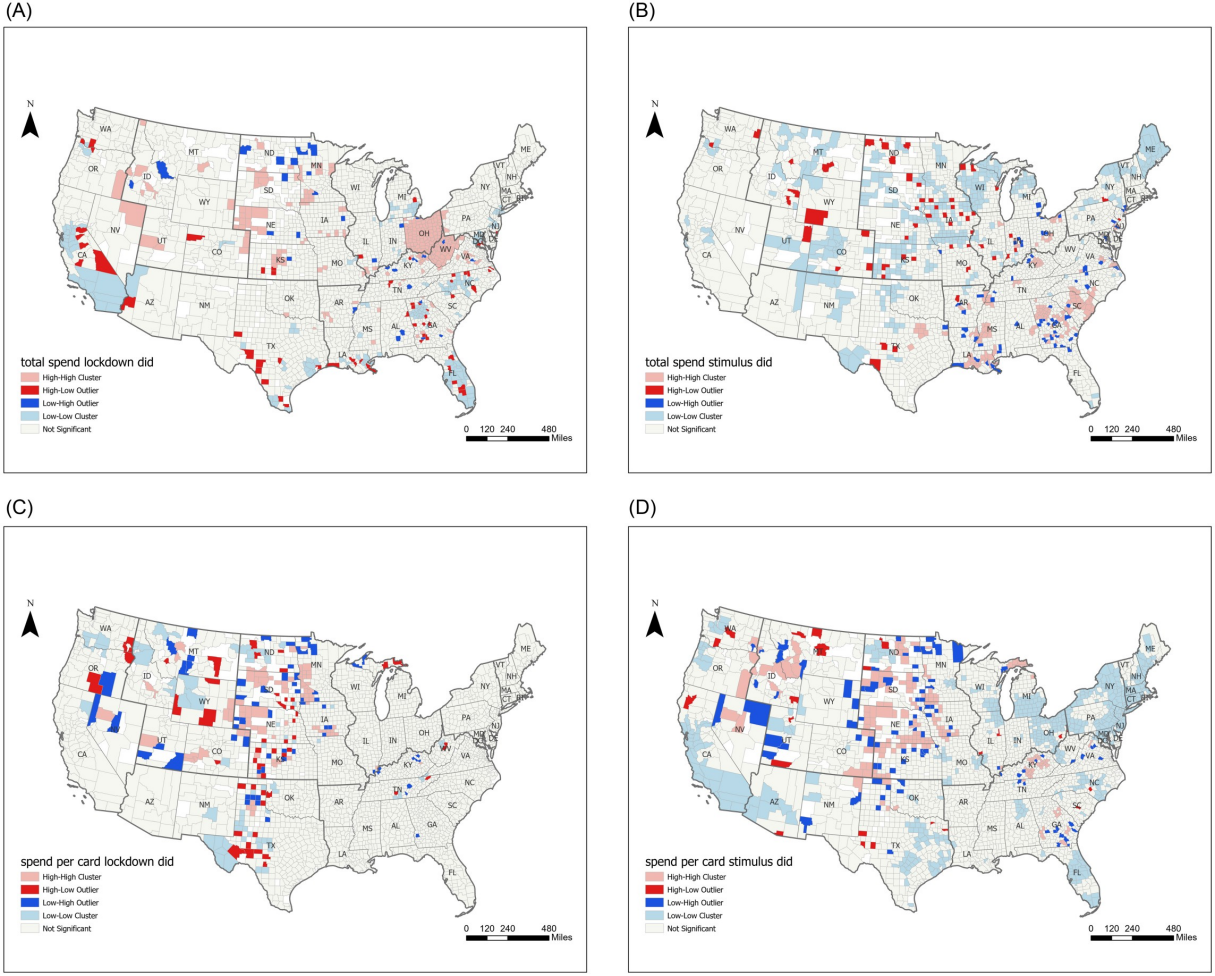
Local Moran’s I spatial association analysis of the spending DID metric, which involves two differences: The first is to subtract the daily average county (or per card) spending in 2020 by the same-day spending in 2019; and the second is to subtract this year-over-year dollar change after each treatment by that before each treatment (the lockdowns or stimulus payments, respectively). (a) Local Moran’s I spatial statistic for county spending DID metric (After Lockdown). (b) Local Moran’s I spatial statistic for county spending DID metric (After Stimulus). (c) Local Moran’s I spatial statistic for per card spending DID metric (After Lockdown). (d) Local Moran’s I spatial statistic for per card spending DID metric (After Stimulus).

We then use the local Moran’s I statistic [45] to further examine the high/low spatial association patterns among neighbouring counties. As shown in Fig 3, after the initial lockdown, there emerged many local clusters with great declines (i.e., the low-low clusters on the map) in New York, New Jersey, Maryland, Michigan, California, Florida, Texas, Georgia, North Carolina, Louisiana, and so on. Many of those local clusters with great spending declines emerged in the hot-spot regions with surging COVID-19 cases in late March [56, 58]. After the stimulus payments, most of the local high-high clusters of spending rebounds emerged in the Southeast region. This is consist with our earlier DID regression analysis at the zip code level by economic regions, although leveraging the local spatial association analysis has allowed us to further identify the spatial variations even within the same economic region and also the spatial dependency among neighboring counties across state or region borders. Regarding the per-card analysis, the low-low clusters of great declines upon the initial lockdown emerged mostly in Far West, Rocky Mountains, and Southwest, as well as in Texas, while the high-high clusters of large rebounds after the stimulus payments mostly appeared in Plains and Rocky Mountains.

Overall, these analyses reveal that different geographic areas exhibited varied levels of needs and urgency for the stimulus program since the onset of the pandemic. Also importantly, the stimulus program generated differential effectiveness in stimulating spending across geographic areas, hence unveiling the imperative need for more geo-targeted stimulus programs.

### RQ3: Impacts of lockdowns and stimulus payments on spending across categories

**Fig 4:**
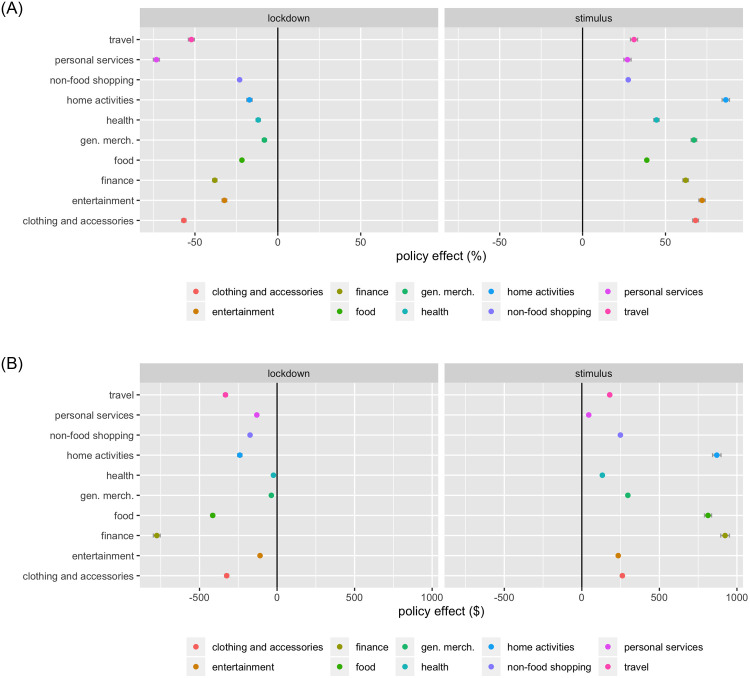
Effects of initial lockdown and stimulus payments by major spending group. (a) Effects on percentage change. (b) Effects on dollar change.

**Fig 5:**
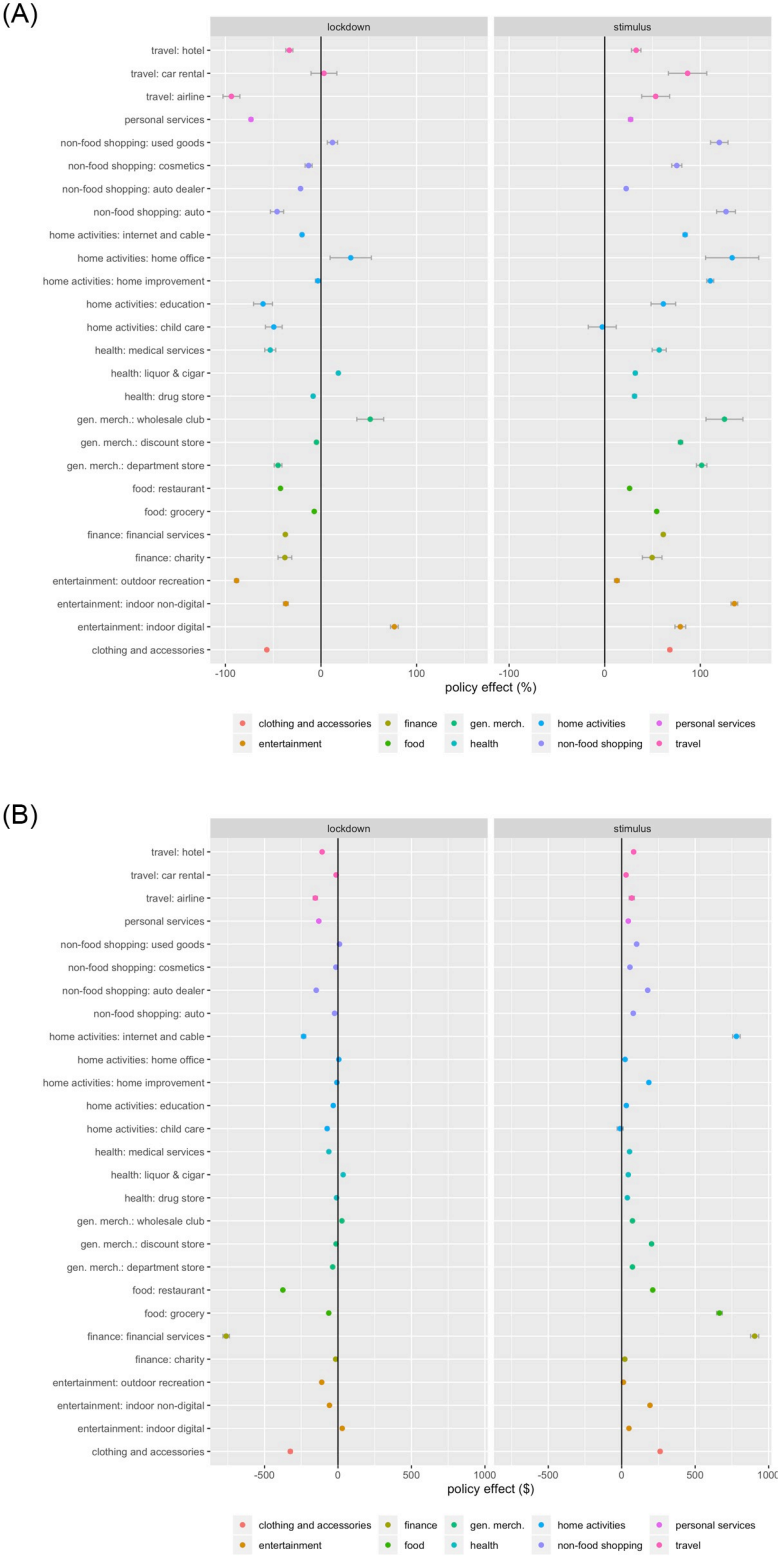
Effects of initial lockdown and stimulus payments by category. (a) Effects on percentage change. (b) Effects on dollar change.

Figs 4 and 5 visualize the percentage change and dollar change after the initial lockdown and stimulus payments by major spending groups and across categories, respectively. The left panel of each graph depicts the *β* estimates (lockdown) and the right panel *γ* estimates (stimulus payments). S4-S108 Tables in S1 File respectively exhibit the parameter estimates of the overall effects, and effects by political affiliations and regions, on the percentage change and dollar change for each major spending group and the key categories within each group. Below, we will first discuss the impacts of the lockdown and stimulus payments on the percentage change, which is more comparable across major spending groups and across categories, then on the dollar change.

#### Percentage change after lockdown

The left panels of Figs 4a and 5a visualize the impact of the initial lockdown on the percentage changes across major spending groups and their categories, respectively. A few key findings emerge: (1) All major spending groups exhibited declines, varying from a reduction of 8.094 (general merchandise) to 73.247 (personal service) percentage points. (2) The declines were more pronounced among the less essential, or more hedonic, categories, particularly personal services (e.g., spa, salon) with a decrease of 73.247 percentage points, clothing and accessories (56.691), travel (52.151; e.g., airlines, cruising), and entertainment (32.198; particularly outdoor recreation like sports and parks: 88.356). (3) Another category with the greatest decline (38.025) was finance related spending (e.g., financial, charity, employment services), potentially as a result of the wide spread perception of the health and financial risks entailed by the pandemic. (4) The top categories witnessing the least decline include general merchandise, health (11.822), and home activities (17.094). (5) Great heterogeneity also emerged across categories within the same major spending group. For instance, even in those categories with major declines, such as travel, the category of car rental experienced an increase (3.076 percentage points), likely due to the public’s reluctance to take public transportation. Similarly, within entertainment, indoor digital entertainment (such as gaming and movies) experienced an increase of 76.78 percentage points. Also, while grocery spending slightly decreased (7.053), the decline in food spending (21.651) stemmed primarily from the reduced spending towards restaurants (42.387), potentially due to their health risks and lockdown closures. (6) Among the major spending groups with the least declines, two types of categories actually saw increases: the cost-saving categories (used goods: 12.002; wholesale clubs: 51.534) and home office category (31.127). Liquor and cigar stores experienced an increase as well (18.136), potentially reflecting the increased anxiety over the pandemic and stress introduced by stay-at-home, job loss, child care, among others.

In summary, the spending declines were apparent after the pandemic commenced, but displayed great heterogeneity across categories. Also, the spending has largely transitioned from outdoor to indoor activities (such as home office, home entertainment), and towards more economical alternatives (such as used goods) in anticipation of a prolonged economic downturn and potential unemployment. A number of concerning observations also emerge, such as the increased spending on liquor and cigar, and decreased spending towards heath, financial and employment services. These findings also corroborate with the prior research that examines consumer behavior under emotional stress. For instance, consumers experiencing a stressful situation would strategically allocate their resources to gain control of their environment, by increasing savings (thus reducing spending), or increasing spending towards necessity products (such as car rental and home office in our context) [59]. Social exclusion, isolation, or loneliness resulting from, for instance, the lockdowns, may also contribute to increased materialism and spending on material goods [60]. Social exclusion may also lead consumers to buying more products symbolic of group membership (such as social gaming or liquor for group consumption in our context) [61].

#### Percentage change after stimulus payments

After the stimulus payments, the following key findings arise: (1) All categories displayed rebounds, varying from an increase of 27.074 (personal service) to 86.363 (home activities) percentage points. (2) Home activities (86.363) and entertainment (72.069) exhibited the highest increases, followed by clothing and accessories (68.147), and general merchandise (67.158). (3) The stimulus payments also mitigated the decline in financial, charity, and employment services (62.169), as well as health (44.573)—the two categories that exhibited concerning declines upon the initial lockdown. (4) The percentage change across all categories rebounded after the stimulus payments, except for child care (with a reduction of 2.57 percentage points), potentially because the COVID health risks left families with limited childcare options; or stay-at-home or unemployed parents chose to home-care or home-school their children. (5) Among the top-increasing categories discussed earlier, three groups of categories stood out: home office (133.344) and home improvement (110.444), indoor non-digital entertainment (135.663; such as toys, books, craft), and more economic categories (such as used good 119.888; wholesale clubs 125.289). The increased spending on home improvement is consistent with the latest media coverage and partly attributable to the increased time at home [62]. This observation can also be explained by the prior consumer behavior research, which suggests that consumers engage in more self-enhancement when facing life constraints [28–30]. The large rebound of the cosmetics category observed in our data, another important aspect of consumers’ self-enhancement, also corroborates with this theory. (6) While essential categories, including health (medical services), food, financial, charity, and employment services experienced declines after the initial lockdown, some even to a serious extent, the stimulus payments mitigated these declines. Specifically, medical services saw an increase of 56.999 percentage points, food 38.729, and financial, charity, and employment services 62.169.

Overall, the stimulus payments offered an important policy mitigation that curbed the initial spending declines, particularly in those major spending groups and categories essential to the low-income population’s health (medical services, grocery) and economic recovery (finance). They also potentially reduced the digital divide across the low- versus high-income populations, with escalating spending on Internet, cable, and telecommunication by the low-income population [63]. Nonetheless, the stimulus payments also elevated the less essential categories (indoor digital entertainment, travel) and even a few concerning categories, such as liquor and cigar, therefore suggesting room for potential improvement of the stimulus program, such as via category-specific or category-targeted stimulus plans, or consumption vouchers implemented by other governments [64, 65].

#### Dollar changes after the lockdown and stimulus payments

Compared to the percentage changes, the dollar changes revealed a similar overall spending pattern (Figs 4b and 5b). Nonetheless, some categories with the largest percentage declines, such as personal service and clothing, actually experienced relatively small dollar declines ($130.514 and $324.618 respectively), potentially as they reflected a small portion of an individual’s overall spending. On the other hand, a few categories, such as food ($414.792), financial, charity, and employment services ($775.464), while moderately declined in the percentage change, accounted for large dollar declines. Then, after the stimulus payments were distributed, although financial, charity, and employment services exhibited a moderate percentage increase as compared to other categories, they represented the largest increase in dollar amount ($923.597). Similarly, grocery, not a top category in percentage increase, emerged on top in dollar increase ($665.329). This is of important implication to resolving food insecurity that became more wide spread across the nation after the onset of the pandemic [66]. Lastly, spending on Internet, cable, and telecommunication, ranked high on not only the percentage increase (84.121%), but also dollar increase ($780.411), may have captured the elevated need of staying connected with work, families, friends, and home entertainment. This increased investment by the low-income population on Internet and cable points to a positive potential of closing the digital divide in the country. Overall, we observe a positive dollar trend with those categories reflecting the essential needs during the public health and economic crisis—food security, financial security, and digital connectedness, which are also closely related to work productivity, social engagement, and mental health.

Further discussions regarding the cross-category comparisons by political affiliation and by region are provided as follows.

#### Spending changes across categories and zip codes of different political affiliations

Overall, the effects by zip codes of different political affiliations for each category remain consistent with those when pooling all categories together. That is, compared to the Republican zip code, the democratic zip codes exhibited a more volatile shift, with a more dramatic decline in the dollar change after the initial lockdown, and then a larger rebound after the stimulus payments, albeit a smaller rebound in the percentage change. A few categories, nonetheless, exhibited interesting, minor deviations from this primary result.

For instance, in the travel category of *car rental*, spending actually increased initially; and this increase was driven by the Republican zip codes’ increased dollar change ($8.961) and percentage change (102.836 percentage points). In contrast, the Democratic zip codes reduced the dollar change ($19.577) and percentage change (26.985) after the initial lockdown. Although the Democratic zip codes saw positive dollar change ($31.175) and percentage change (93.754) after the stimulus payments, their net dollar change and percentage change remained much smaller than those of the Republican zip codes. In another category, *outdoor recreation*, the Democratic zip codes showed less rebound in both the dollar change ($9.775) and percentage change (6.393), relative to the Republic zip codes ($13.057 and 18.763 respectively), consistent with the prior research on the heightened risk perception among the Democratic than Republican areas [52–55].

Conversely, in the entertainment category of *in-home digital entertainment*, the Democratic zip codes displayed a greater increase in the dollar change both after the lockdown ($36.567) and stimulus payments ($60.322), compared to their Republic counterparts ($21.424 and $38.986 respectively). The Democratic zip codes also exhibited a higher net percentage change (157.439) than the Republican zip codes (154.685). Finally, the Democratic zip codes also saw more increase in the *cigar and liquor*’s dollar change both after the lockdown ($48.155) and stimulus payments ($61.805), compared to the Republic zip codes ($22.621 and $27.378 respectively). The resulting net percentage change after the stimulus payments turned out to be an increase of 52.119 percentage points for the Democratic zip codes and 48.143 for the Republican zip codes.

Overall, the Republic zip codes drove the spending increase in car rental and rebound in outdoor recreations; whereas the Democratic zip codes drove the increases in indoor digital entertainment, as well as cigar and liquor.

#### Spending changes across categories and economic regions

The category-specific analyses further revealed the spatial heterogeneity across the eight economic regions. We will discuss below, as examples, such heterogeneity in the dollar change among a few major spending groups and categories of importance to the low-income population’s well-being. More detailed results for each category and each geographic region can be found in the S4-S108 Tables in S1 File.

*Travel*. Mideast experienced the largest initial and overall declines in the travel dollar change after the initial lockdown ($433.777) and stimulus payments ($249.71), respectively. In the *airline* category, Great Lakes experienced the most pronounced initial ($248.759) and overall ($145.5) declines. In another category of *car rental*, New England showed the strongest increase ($36.705) after an initial minor decline then a major rebound after the stimulus payments.

*Home—internet, cable, and telecommunication*. Southeast experienced the greatest initial decline ($324.799) and Rocky Mountains the least ($55.812). After the stimulus payments, New England had the least overall gain ($248.722), whereas Great Lakes the greatest ($728.927).

*Health*. Far West experienced the largest initial drop in the health spending amount ($38.115), whereas Rocky Mountains saw no statistically significant effect. The overall spending gain after the stimulus payments was the largest for Great Lakes ($147.828) and least for New England ($59.559). In the *liquor and cigar* category, Far West exhibited the least initial increase ($3.677) and Southwest the most ($54.859). The overall gain after the stimulus payments was the lowest at Far West ($42.803) and highest at Great Lakes ($108.604).

*Food—grocery*. Mideast displayed a large initial gain in grocery spending ($89.520) whereas Southwest had a huge dip ($247.960) initially after the lockdown and then a large increase ($952.408) after the stimulus payments, Overall, Great Lakes had the largest overall gain in the grocery spending ($821.404).

*Entertainment*. Far West showed the largest initial decline ($133.735) and the least overall gain ($38.867) in entertainment, particularly in the *outdoor recreation* category. In contrast, Great Lakes showed the largest overall gain ($207.411) after the stimulus payments. In the *outdoor recreation* category, Plains exhibited the least overall reduction ($54.603). In the *indoor digital entertainment* category, Southwest gained the most initially ($36.408) and Mideast had the largest net gain after the stimulus payments ($93.261).

*Finance*. Southwest showed the largest initial decline ($1031.536) whereas New England the least ($453.322). After the stimulus payments, Mideast displayed an overall dip of $273.515. In contrast, Southeast gained $449.561.

The category-by-category and region-based analyses reveal that Southeast experienced the greatest gain in financial, charity, and employment services. Far West had the least gain over entertainment, cigar and alcohol. In addition, New England showed the largest overall gain in car rental, yet least in health, Internet, cable, and telecommunication. Mideast showed the most gain in indoor digital entertainment and most loss in travel, particularly, hotel, and financial, charity, and employment services. Great Lakes had the most cut on airline, yet the highest overall lift on entertainment, grocery, Internet, cable, and telecommunication, as well as cigar and alcohol. In short, these results depict a more granular picture of the category-specific, spatially heterogeneous impacts on the low-income population’s spending during the crisis.

#### Summary of answers to research questions

Below we summarize the key findings revolving around the four key research questions raised earlier.

RQ1: Inter-temporally, the state lockdowns diminished the daily average spending relative to the same period in 2019 by $3.9 per card and $2,214 per zip code, whereas the stimulus payments elevated the daily average spending by $15.7 per card and $3,307 per zip code.
RQ2: The spatial heterogeneity prevailed. Southwest exhibited the highest initial decline in spending after the lockdowns, whereas Southeast had the largest net gain after the stimulus payments. Also, Democratic zip codes displayed much more volatile dynamics, with an initial decline three times that of Republican zip codes, followed by a higher rebound and a net gain after the stimulus payments. In addition, the local spatial association analysis identified those local clusters with great spending declines in the hot-spot regions with surging COVID-19 cases in late March 2020; after the stimulus payments, most of the local high-high clusters of spending rebounds emerged in Southeast.
RQ3: Across 26 categories, the stimulus payments promoted spending in those categories that enhanced public health and charitable donations, reduced food insecurity and digital divide, while having also stimulated non-essential and even undesirable categories, such as liquor and cigar.
RQ4: Our results unveil strong geo- and cross-category heterogeneities, and thus an imperative need for more geo- and category-targeted stimulus programs, as well as more strategic policy communications, to protect and promote equity and well-being of the low-income population during crises.

## Discussion and conclusion

This research demonstrates that the COVID-19 pandemic entailed a strong economic impact on the low-income population’s spending in the U.S., over time, across geographic areas, and across spending categories, particularly among the Democratic areas, as well as Far West and New England where the early COVID-19 hot spots arose. The stimulus program largely curbed the initial spending declines and stimulated the spending across a number of major spending groups and categories of essential importance to this population’s well-being. For instance, the rebound and net increase in the grocery spending helped mitigate the increasingly grave food insecurity in the U.S. The stimulus payments also negated the initial decrease in the spending on medical services critical amid the pandemic. Another positive impact is the elevated spending towards the Internet, cable, and telecommunication, potentially reducing digital divide and social disparity with significant implications for equitable access to WiFi, tele-medicine, online education, and others. Furthermore, the rebound and elevated overall spending on financial, charity, and employment services is instrumental to the economic recovery among the low-income population.

Nonetheless, the stimulus payments also stimulated non-essential categories and even undesirable categories, such as liquor and cigar, among the low-income population. Thus, this uniform program can be further improved with sharper geo- and category-targeting, for instance, (a) targeting the areas hit the hardest or the earliest by the crisis, with the greatest health risks, highest unemployment rate, or the most volatile spending shifts, and (b) targeting the most essential categories of top importance to the low-income population’s physical, mental, and financial health, such as grocery and medical, as accomplished by the category-specific stimulus vouchers [64, 65]. Earlier studies have indeed demonstrated that different forms of stimulus programs vary in their effectiveness in stimulating spending. For instance, the 2009 reduction in withholding boosted spending at roughly half the rate as the 2008 payments [67]. Hence, various improvements of the stimulus programs have been proposed, such as targeting the individuals with the highest Marginal Propensity to Consume [36], or enlisting social insurance [38], voluntary loans [68], or coupons [69]. Our research thus enriches these policy recommendations with geo-spatial and cross-categorical dimensions, particularly in light of the distinct nature of the convoluted public health and economic crisis. Another crucial element to accompany an effective stimulation program is more targeted and strategic policy communications. For instance, more guided policy communications could be leveraged to direct spending towards more essential categories, such as grocery and medical, and away from less essential or more hedonic (and potentially harmful) categories, such as gaming, liquor and cigar, as observed in our data. Such policy communications may also help mitigate the core crisis (COVID-19 spread) by, for instance, promoting grocery or restaurant deliveries over dine-ins, outdoor over indoor entertainment, family, social time, learning and self-improvements (home office, education, Internet) instead of liquor and cigar consumption to support mental health. Such communications should also advocate continued efforts towards seeking re-employment or continued spending toward employment services.

Lastly, our findings shed valuable lights on broader economic and business policies and strategies. For instance, the analyses offer empirical evidence of the importance of a business model’s (and more broadly an economy’s) crisis preparedness, resilience, and capability for agile transformation and accelerated economic recovery. Also, at a time of a convoluted public health and economic crisis, the low-income population’s spending has apparently leaned even more towards the more economical options (such as wholesale clubs, used goods, and cheaper brands), indoor/home options (home office, home entertainment), and digital options (such as digital entertainment). Industries or economies relying predominantly on offline or non-digital income (such as theaters, sports stadiums, and hotels) hence need to accelerate digital transformation and resilience design. Moreover, this pandemic has profoundly influenced the population’s work and life styles, such as working from home, tele-conferencing, tele-medicine, online education, grocery delivery, residential choice, thus leading to broad and long-term impacts on the competitive landscape across many industries and a nation’s economic structure. The low-income population’s shifting spending patterns also provide important guidance to broad advertising and communication strategies, pointing to the imperative need to empathetically align communications with consumers’ focus on, such as life, health, and social equality, to support and accelerate business and economic recovery.

In summary, this research investigates the inter-temporal, joint impacts of the two essential government mitigation policies amid the unprecedented public health and economic crisis, the lockdowns and stimulus payments, on the daily expenditures of one of the most vulnerable populations in the U.S., the low-income population. The granular spatial-temporal analyses and comprehensive cross-categorical analyses also reveal strong heterogeneities across geography and spending categories, thus recommending more geo- and category-targeted mitigation policies to protect and promote the well-being of the low-income population that is of essential importance to social equality and economic recovery.

Despite the contributions, this research presents limitations and hence calls for future research. For instance, it would be interesting to develop a deeper understanding of the online versus offline spending substitution during the crisis, rendering additional insights regarding channel migration and digital transformation among this vulnerable population. Another interesting direction is to explore the dynamic spending patterns at the individual level, when more granular data become available documenting the specific date when receiving the stimulus check, details of individual transactions, and demographics at individual level.

## Supporting information

S1 File Supplementary material: S1 and S2 Figs and S1-S108 Tables. (PDF)
