# Supplementary material for: Impacts of COVID-19 lockdowns and stimulus payments on low-income population’s spending in the United States
Source: PLoS One. 2021 Sep 8;16(9):e0256407. doi: 10.1371/journal.pone.0256407 (PMC8425560; doi:10.1371/journal.pone.0256407)
Supplement: S1 File — (PDF) [file pone.0256407.s001.pdf]

Supplementary Material

Figures

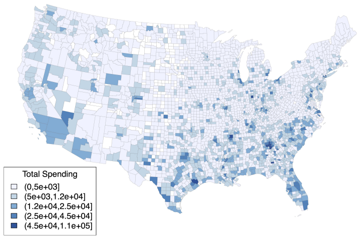

(a) Before Lockdown 2020

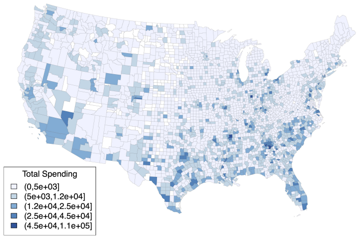

(b) After Lockdown 2020

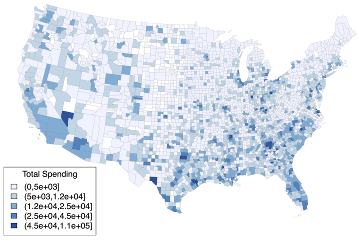

(c) After Stimulus 2020

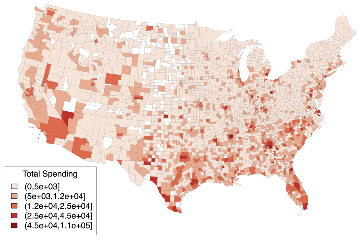

(d) Before Lockdown 2019

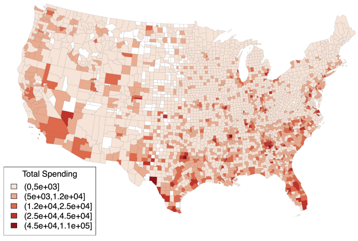

(e) After Lockdown 2019

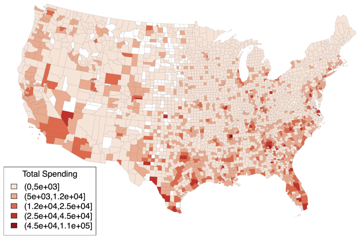

(f) After Stimulus 2019

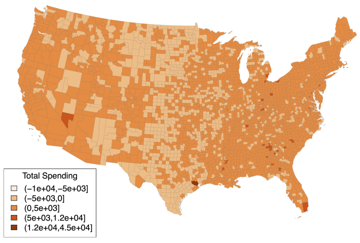

(g) Before Lockdown 2020 - 2019

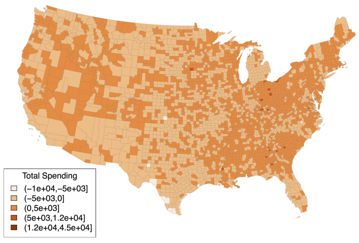

(h) After Lockdown 2020 - 2019

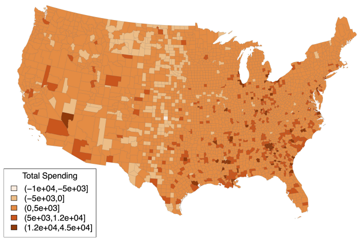

(i) After Stimulus 2020 - 2019

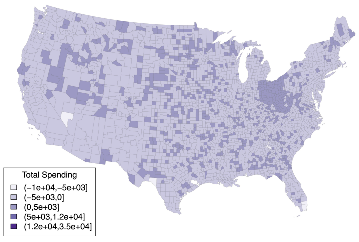

(j) After Lockdown DID

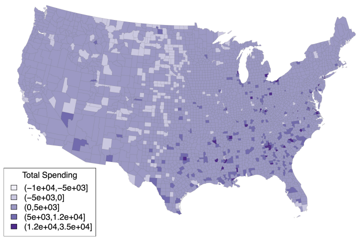

(k) After Stimulus DID

S1 Fig: Spatial distribution of daily average zip code spending over different periods.

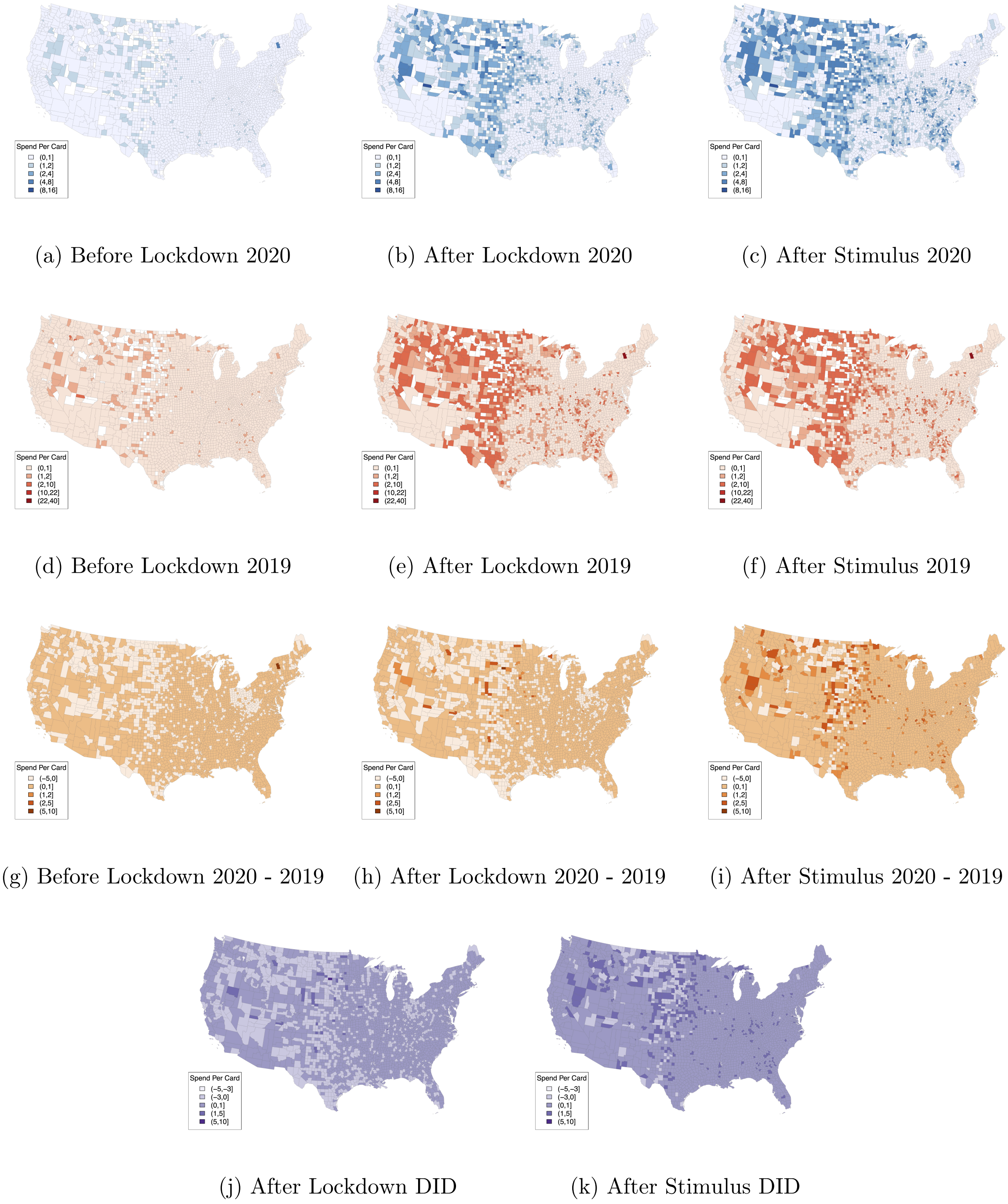

(a) Before Lockdown 2020 (b) After Lockdown 2020 (c) After Stimulus 2020

(d) Before Lockdown 2019 (e) After Lockdown 2019 (f) After Stimulus 2019

(g) Before Lockdown 2020 - 2019 (h) After Lockdown 2020 - 2019 (i) After Stimulus 2020 - 2019

(j) After Lockdown DID (k) After Stimulus DID

S2 **Fig**: Spatial distribution of daily average per card spending over different periods.

Tables

S1 Table: Major Spending Groups and Categories.

Spending Group	Category	Sub-category
Travel	airlines<br>car rental<br>hotels	
Home Activities	internet	internet, cable, telecommunication, etc
	home office	Computer maintenance, e-repair, office stores, etc
	home improvement	Glassware, air-condition, furniture, floor-covering, etc
	education	school tuition, educational services
	child care	
Health	medical services	physician, dental, opticians, nursing, osteopaths, etc
	liquor & cigar stores	beer, wine, tobacco
	drug stores	pharmacies
General merchandise	wholesale clubs	e.g. Costco
	department stores	e.g. Macy's
	discount stores	e.g. dollar tree
Food	restaurants	catering, restaurant, drinking, fast-food
	grocery	grocery store, freezer candy, nuts, dairy, bakery
Entertainment	outdoor recreation	movie, band, orchestra, dance hall, tourist, betting, park, club, aquariums, etc
	indoor digital	game, book, movies, music, online gambling
	indoor non-digital	toys, books, craft, photography, etc
Other non-food Shopping	used goods	antique, pawn, etc
	cosmetics	e.g. Sephora
	auto dealers	car selling/leasing, accessories stores, etc
	auto	auto repair, tire, paint, car wash, towing, etc
Finance		tax, real estate, charity, political fundraising, employment services, etc
Personal Services		dryer, dating, funeral, spa, massage, tatto, shoe repair, etc
Clothing and Accessories		jewelry, clocks, silverware, clothes, fur, shoe, tailor, etc

.1 Daily Zip Code Level Spending

S2 Table: Effects of staggered lockdowns and stimulus payments on zip code level spending.

	Dollar Change ($)			Percentage Change (%)		
	All	Republican	Democratic	All	Republican	Democratic
$\beta : \mathbb{1}\{\geq order\_date\}$	-1689.657***	-686.725***	-2890.987***	-19.503***	-15.945***	-24.182***
	(36.548)	(25.792)	(71.184)	(0.282)	(0.412)	(0.360)
$\gamma : \mathbb{1}\{\geq 04-11\}$	3409.338***	2383.852***	5085.928***	39.386***	41.633***	35.325***
	(44.855)	(35.932)	(100.879)	(0.240)	(0.318)	(0.358)
Adjusted R^2	0.467	0.419	0.482	0.223	0.215	0.240
N	2521355	1589995	931360	2521355	1589995	931360

***** 0.01 **0.05 * 0.1**

Note: The regressions include the zip-month fixed effects. The standard errors shown in the parenthesis are clustered at the zip code level.

S3 Table: Global Moran's I statistical analysis for DID of daily average county and per card spending after lockdown and stimulus payments.

Title	Period	Moran's Index	Z-value	Results
County Spending DID	after lockdown	0.019	5.202	clustered
	after stimulus	0.115	12.813	clustered
Per Card Spending DID	after lockdown	0.100	9.098	clustered
	after stimulus	0.252	22.435	clustered

.2 Travel Spending

Total

S4 Table: Effects of initial lockdown and stimulus payments on zip code level travel spending

	Dollar Change ($)			Percentage Change (%)		
	All	Republican	Democratic	All	Republican	Democratic
$\beta : \mathbb{1}\{\geq 03 - 19\}$	-332.649***	-177.101***	-476.996***	-52.151***	-45.187***	-58.613***
	(5.880)	(4.342)	(10.272)	(0.928)	(1.484)	(1.139)
$\gamma : \mathbb{1}\{\geq 04 - 11\}$	179.719***	124.114***	228.512***	31.037***	38.332***	24.636***
	(4.351)	(4.077)	(7.288)	(1.100)	(1.889)	(1.225)
Adjusted R^2	0.224	0.118	0.259	0.144	0.131	0.151
N	1245420	601982	643438	1245420	601982	643438

***** 0.01 **0.05 * 0.1**

Note: The regressions include the zip-month fixed effects. The standard errors shown in the parenthesis are clustered at the zip code level.

S5 Table: Effects on travel dollar change across geographic regions

	Dependent Variable: Dollar Change ($)							
	New England	Mideast	Southeast	Great Lakes	Plains	Rocky Mountains	Southwest	Far West
$\beta : \mathbb{1}\{\geq 03 - 19\}$	-238.813***	-433.777***	-317.248***	-367.296***	-210.460***	-266.089***	-297.472***	-359.186***
	(17.775)	(21.401)	(10.407)	(17.512)	(13.673)	(19.837)	(12.877)	(13.842)
$\gamma : \mathbb{1}\{\geq 04 - 11\}$	127.277***	184.067***	203.001***	244.026***	124.936***	95.401***	163.951***	134.610***
	(12.730)	(13.568)	(7.580)	(13.516)	(10.937)	(21.983)	(11.837)	(9.686)
Adjusted R^2	0.152	0.248	0.266	0.157	0.118	0.116	0.248	0.181
N	60057	192585	366219	176632	83371	41011	153891	172635

***** 0.01 **0.05 * 0.1**

Note: The regressions include the zip-month fixed effects. The standard errors shown in the parenthesis are clustered at the zip code level.

S6 Table: Effects on travel percentage change across geographic regions

	Dependent Variable: Percentage Change (%)							
	New England	Mideast	Southeast	Great Lakes	Plains	Rocky Mountains	Southwest	Far West
$\beta : \mathbb{1}\{\geq 03 - 19\}$	-68.551***	-62.882***	-48.942***	-55.268***	-48.852***	-50.949***	-38.168***	-53.929***
	(5.227)	(2.422)	(1.711)	(2.485)	(3.990)	(5.225)	(2.300)	(2.280)
$\gamma : \mathbb{1}\{\geq 04 - 11\}$	28.415***	18.480***	39.435***	46.036***	37.778***	29.837***	24.246***	17.585***
	(5.512)	(2.456)	(2.171)	(2.972)	(4.946)	(6.425)	(2.949)	(2.541)
Adjusted R^2	0.152	0.158	0.137	0.141	0.139	0.138	0.138	0.141
N	60057	192585	366219	176632	83371	41011	153891	172635

***** 0.01 **0.05 * 0.1**

Note: The regressions include the zip-month fixed effects. The standard errors shown in the parenthesis are clustered at the zip code level.

Airlines

S7 Table: Effects of initial lockdown and stimulus payments on zip code level airlines spending

	Dollar Change ($)			Percentage Change (%)		
	All	Republican	Democratic	All	Republican	Democratic
$\beta : \mathbb{1}\{\geq 03-19\}$	-154.360***	-90.181***	-171.610***	-93.717***	-82.489***	-96.734***
	(7.332)	(9.397)	(8.838)	(4.526)	(11.634)	(4.813)
$\gamma : \mathbb{1}\{\geq 04-11\}$	68.360***	58.402***	70.449***	53.313***	68.883***	50.046***
	(8.919)	(20.041)	(9.932)	(7.451)	(20.922)	(7.879)
Adjusted R^2	0.181	0.210	0.176	0.189	0.232	0.173
N	106376	23980	82396	106376	23980	82396

***** 0.01 **0.05 * 0.1**

Note: The regressions include the zip-month fixed effects. The standard errors shown in the parenthesis are clustered at the zip code level.

S8 Table: Effects on airlines dollar change across geographic regions

	Dependent Variable: Dollar Change ($)							
	New England	Mideast	Southeast	Great Lakes	Plains	Rocky Mountains	Southwest	Far West
$\beta : \mathbb{1}\{\geq 03-19\}$	-106.142***	-192.843***	-145.844***	-248.759***	-143.143***	-124.428***	-127.092***	-105.301***
	(30.961)	(20.008)	(13.574)	(27.966)	(27.540)	(22.361)	(13.821)	(14.230)
$\gamma : \mathbb{1}\{\geq 04-11\}$	3.541	97.818***	71.356***	103.259***	2.806	37.079	38.752*	60.039***
	(46.159)	(28.041)	(17.890)	(22.852)	(48.160)	(48.145)	(21.362)	(17.035)
Adjusted R^2	0.198	0.200	0.169	0.149	0.216	0.153	0.217	0.157
N	3158	15987	26837	13955	4276	4276	16888	21074

***** 0.01 **0.05 * 0.1**

Note: The regressions include the zip-month fixed effects. The standard errors shown in the parenthesis are clustered at the zip code level.

S9 Table: Effects on airlines percentage change across geographic regions

	Dependent Variable: Percentage Change (%)							
	New England	Mideast	Southeast	Great Lakes	Plains	Rocky Mountains	Southwest	Far West
$\beta : \mathbb{1}\{\geq 03-19\}$	-107.477***	-107.520***	-88.736***	-118.338***	-88.262***	-118.630***	-81.075***	-75.743***
	(23.551)	(10.075)	(8.632)	(10.190)	(23.634)	(23.128)	(13.677)	(10.816)
$\gamma : \mathbb{1}\{\geq 04-11\}$	31.691	91.739***	45.747***	64.763***	30.777	9.161	29.511	56.978***
	(38.138)	(17.667)	(14.221)	(15.923)	(39.309)	(38.597)	(24.938)	(16.219)
Adjusted R^2	0.240	0.175	0.184	0.190	0.244	0.205	0.178	0.184
N	3158	15987	26837	13955	4276	4276	16888	21074

***** 0.01 **0.05 * 0.1**

Note: The regressions include the zip-month fixed effects. The standard errors shown in the parenthesis are clustered at the zip code level.

Car Rental

S10 Table: Effects of initial lockdown and stimulus payments on zip code level car rental spending

	Dollar Change ($)			Percentage Change (%)		
	All	Republican	Democratic	All	Republican	Democratic
$\beta : \mathbb{1}\{\geq 03-19\}$	-12.969***	8.961**	-19.577***	3.076	102.836***	-26.985***
	(2.350)	(4.360)	(2.746)	(6.896)	(16.688)	(7.373)
$\gamma : \mathbb{1}\{\geq 04-11\}$	29.436***	21.719***	31.175***	86.714***	55.473*	93.754***
	(3.232)	(6.459)	(3.682)	(10.245)	(29.777)	(10.609)
Adjusted R^2	0.096	0.133	0.086	0.137	0.172	0.122
N	355734	84199	271535	355734	84199	271535

***** 0.01 **0.05 * 0.1**

Note: The regressions include the zip-month fixed effects. The standard errors shown in the parenthesis are clustered at the zip code level.

S11 Table: Effects on car rental dollar change across geographic regions

	Dependent Variable: Dollar Change ($)							
	New England	Mideast	Southeast	Great Lakes	Plains	Rocky Mountains	Southwest	Far West
$\beta : \mathbb{1}\{\geq 03-19\}$	-6.280	-25.978***	-10.139**	-31.340***	14.359	-2.728	-4.477	-9.965**
	(10.382)	(5.874)	(5.111)	(7.781)	(9.034)	(11.777)	(5.842)	(4.378)
$\gamma : \mathbb{1}\{\geq 04-11\}$	42.985**	32.262***	40.944***	53.832***	12.892	-5.882	-0.799	20.131***
	(17.065)	(7.747)	(6.954)	(10.135)	(13.264)	(15.386)	(7.716)	(6.114)
Adjusted R^2	0.115	0.103	0.092	0.109	0.111	0.104	0.098	0.079
N	16161	58713	86254	44501	15993	11360	49376	73400

***** 0.01 **0.05 * 0.1**

Note: The regressions include the zip-month fixed effects. The standard errors shown in the parenthesis are clustered at the zip code level.

S12 Table: Effects on car rental percentage change across geographic regions

	Dependent Variable: Percentage Change (%)							
	New England	Mideast	Southeast	Great Lakes	Plains	Rocky Mountains	Southwest	Far West
$\beta : \mathbb{1}\{\geq 03-19\}$	-3.849	-18.667	45.852***	11.194	45.469	-29.520	-0.342	-39.811**
	(34.961)	(15.674)	(13.972)	(20.282)	(35.041)	(43.788)	(16.920)	(15.716)
$\gamma : \mathbb{1}\{\geq 04-11\}$	190.467***	124.968***	72.370***	131.194***	96.326*	-6.235	11.393	70.639***
	(58.471)	(21.055)	(22.286)	(28.974)	(52.861)	(87.337)	(26.660)	(21.489)
Adjusted R^2	0.148	0.144	0.142	0.149	0.155	0.131	0.129	0.113
N	16161	58713	86254	44501	15993	11360	49376	73400

***** 0.01 **0.05 * 0.1**

Note: The regressions include the zip-month fixed effects. The standard errors shown in the parenthesis are clustered at the zip code level.

Hotels

S13 Table: Effects of initial lockdown and stimulus payments on zip code level hotels spending

	Dollar Change ($)			Percentage Change (%)		
	All	Republican	Democratic	All	Republican	Democratic
$\beta : \mathbb{1}\{\geq 03-19\}$	-108.018***	-76.889***	-130.210***	-33.112***	-29.639***	-35.588***
	(3.452)	(4.109)	(5.089)	(1.895)	(3.009)	(2.435)
$\gamma : \mathbb{1}\{\geq 04-11\}$	81.223***	58.514***	95.869***	32.907***	29.124***	35.347***
	(4.048)	(5.024)	(5.796)	(2.539)	(4.199)	(3.180)
Adjusted R^2	0.200	0.109	0.225	0.125	0.135	0.118
N	431098	177674	253424	431098	177674	253424

***** 0.01 **0.05 * 0.1**

Note: The regressions include the zip-month fixed effects. The standard errors shown in the parenthesis are clustered at the zip code level.

S14 Table: Effects on hotels dollar change across geographic regions

	Dependent Variable: Dollar Change ($)							
	New England	Mideast	Southeast	Great Lakes	Plains	Rocky Mountains	Southwest	Far West
$\beta : \mathbb{1}\{\geq 03-19\}$	-66.959***	-117.128***	-130.423***	-130.567***	-62.155***	-64.311***	-74.074***	-105.558***
	(14.379)	(13.180)	(6.065)	(9.804)	(10.507)	(14.223)	(8.175)	(8.462)
$\gamma : \mathbb{1}\{\geq 04-11\}$	30.597*	68.623***	96.476***	131.934***	63.773***	13.381	47.280***	77.431***
	(17.148)	(11.745)	(7.163)	(11.750)	(14.878)	(17.788)	(10.588)	(9.797)
Adjusted R^2	0.153	0.123	0.288	0.102	0.106	0.089	0.204	0.106
N	10550	45461	142764	53441	23625	16107	72361	66869

***** 0.01 **0.05 * 0.1**

Note: The regressions include the zip-month fixed effects. The standard errors shown in the parenthesis are clustered at the zip code level.

S15 Table: Effects on hotels percentage change across geographic regions

	Dependent Variable: Percentage Change (%)							
	New England	Mideast	Southeast	Great Lakes	Plains	Rocky Mountains	Southwest	Far West
$\beta : \mathbb{1}\{\geq 03-19\}$	-41.326***	-34.136***	-36.692***	-41.688***	-24.831***	-42.015***	-18.852***	-33.483***
	(12.599)	(6.463)	(3.096)	(5.084)	(8.414)	(10.565)	(4.661)	(5.046)
$\gamma : \mathbb{1}\{\geq 04-11\}$	31.226	25.844***	37.582***	48.693***	13.976	12.606	24.526***	33.740***
	(18.962)	(8.022)	(4.073)	(6.921)	(13.417)	(13.783)	(6.487)	(6.496)
Adjusted R^2	0.154	0.134	0.124	0.133	0.133	0.116	0.121	0.113
N	10550	45461	142764	53441	23625	16107	72361	66869

***** 0.01 **0.05 * 0.1**

Note: The regressions include the zip-month fixed effects. The standard errors shown in the parenthesis are clustered at the zip code level.

.3 Home Activities Spending

Total

S16 Table: Effects of initial lockdown and stimulus payments on zip code level home activity spending

	Dollar Change ($)			Percentage Change (%)		
	All	Republican	Democratic	All	Republican	Democratic
$\beta : \mathbb{1}\{\geq 03-19\}$	-240.075***	-132.213***	-405.968***	-17.094***	-16.663***	-17.757***
	(7.048)	(4.992)	(15.954)	(0.833)	(1.141)	(1.176)
$\gamma : \mathbb{1}\{\geq 04-11\}$	870.072***	550.086***	1347.563***	86.363***	88.588***	83.042***
	(13.858)	(9.873)	(30.414)	(1.127)	(1.549)	(1.595)
Adjusted R^2	0.431	0.354	0.443	0.125	0.118	0.137
N	1934896	1167328	767568	1934896	1167328	767568

***** 0.01 **0.05 * 0.1**

Note: The regressions include the zip-month fixed effects. The standard errors shown in the parenthesis are clustered at the zip code level.

S17 Table: Effects on home activity dollar change across geographic regions

	Dependent Variable: Dollar Change ($)							
	New England	Mideast	Southeast	Great Lakes	Plains	Rocky Mountains	Southwest	Far West
$\beta : \mathbb{1}\{\geq 03-19\}$	-110.479***	-273.100***	-336.054***	-231.337***	-87.482***	-65.350***	-238.996***	-168.682***
	(14.079)	(15.081)	(18.240)	(14.088)	(10.692)	(13.582)	(19.216)	(11.135)
$\gamma : \mathbb{1}\{\geq 04-11\}$	417.723***	746.195***	1026.457***	1047.871***	619.765***	507.146***	1105.759***	636.509***
	(29.489)	(32.976)	(28.947)	(44.680)	(36.419)	(31.785)	(35.981)	(26.057)
Adjusted R^2	0.462	0.436	0.401	0.478	0.417	0.247	0.516	0.347
N	94929	277303	590000	307091	169256	61093	224696	212646

***** 0.01 **0.05 * 0.1**

Note: The regressions include the zip-month fixed effects. The standard errors shown in the parenthesis are clustered at the zip code level.

S18 Table: Effects on home activity percentage change across geographic regions

	Dependent Variable: Percentage Change (%)							
	New England	Mideast	Southeast	Great Lakes	Plains	Rocky Mountains	Southwest	Far West
$\beta : \mathbb{1}\{\geq 03-19\}$	-8.703*	-22.041***	-23.847***	-14.408***	-5.862*	-5.351	-14.528***	-15.075***
	(4.675)	(2.345)	(1.470)	(2.215)	(3.022)	(4.564)	(1.951)	(2.306)
$\gamma : \mathbb{1}\{\geq 04-11\}$	79.605***	74.309***	98.406***	106.246***	83.131***	60.663***	76.344***	63.829***
	(5.658)	(3.013)	(1.990)	(3.007)	(4.142)	(6.724)	(2.806)	(2.982)
Adjusted R^2	0.112	0.119	0.122	0.123	0.117	0.126	0.138	0.123
N	94929	277303	590000	307091	169256	61093	224696	212646

***** 0.01 **0.05 * 0.1**

Note: The regressions include the zip-month fixed effects. The standard errors shown in the parenthesis are clustered at the zip code level.

Internet, cable and telecommunication

S19 Table: Effects of initial lockdown and stimulus payments on zip code level Internet, cable and telecommunication spending

	Dollar Change ($)			Percentage Change (%)		
	All	Republican	Democratic	All	Republican	Democratic
$\beta : \mathbb{1}\{\geq 03-19\}$	-233.927***	-129.202***	-384.997***	-19.839***	-19.633***	-20.136***
	(7.179)	(5.193)	(15.663)	(0.810)	(1.113)	(1.157)
$\gamma : \mathbb{1}\{\geq 04-11\}$	780.411***	494.647***	1181.571***	84.121***	83.867***	84.478***
	(13.117)	(9.316)	(27.992)	(1.104)	(1.517)	(1.582)
Adjusted R^2	0.427	0.361	0.433	0.118	0.112	0.129
N	1722910	1014795	708115	1722910	1014795	708115

***** 0.01 **0.05 * 0.1**

Note: The regressions include the zip-month fixed effects. The standard errors shown in the parenthesis are clustered at the zip code level.

S20 Table: Effects on Internet, cable and telecommunication dollar change across geographic regions

	Dependent Variable: Dollar Change ($)							
	New England	Mideast	Southeast	Great Lakes	Plains	Rocky Mountains	Southwest	Far West
$\beta : \mathbb{1}\{\geq 03-19\}$	-117.970***	-267.809***	-324.799***	-239.295***	-94.391***	-55.812***	-200.515***	-161.638***
	(15.092)	(15.171)	(18.949)	(14.679)	(11.325)	(13.359)	(12.990)	(10.563)
$\gamma : \mathbb{1}\{\geq 04-11\}$	366.692***	660.950***	923.030***	968.222***	564.812***	422.227***	965.348***	531.624***
	(27.443)	(29.997)	(27.755)	(42.387)	(35.400)	(28.209)	(31.970)	(23.455)
Adjusted R^2	0.461	0.433	0.404	0.492	0.426	0.247	0.461	0.348
N	77650	239070	541197	266925	142957	53858	207485	195483

***** 0.01 **0.05 * 0.1**

Note: The regressions include the zip-month fixed effects. The standard errors shown in the parenthesis are clustered at the zip code level.

S21 Table: Effects on Internet, cable and telecommunication percentage change across geographic regions

	Dependent Variable: Percentage Change (%)							
	New England	Mideast	Southeast	Great Lakes	Plains	Rocky Mountains	Southwest	Far West
$\beta : \mathbb{1}\{\geq 03-19\}$	-10.105**	-22.812***	-25.822***	-18.387***	-10.409***	-6.676	-18.292***	-17.601***
	(4.519)	(2.316)	(1.409)	(2.183)	(2.960)	(4.956)	(1.900)	(2.290)
$\gamma : \mathbb{1}\{\geq 04-11\}$	76.993***	68.075***	96.735***	104.501***	78.868***	56.585***	79.629***	61.402***
	(5.876)	(2.952)	(1.944)	(2.920)	(3.978)	(6.603)	(2.771)	(3.008)
Adjusted R^2	0.113	0.117	0.115	0.117	0.112	0.111	0.128	0.107
N	77650	239070	541197	266925	142957	53858	207485	195483

***** 0.01 **0.05 * 0.1**

Note: The regressions include the zip-month fixed effects. The standard errors shown in the parenthesis are clustered at the zip code level.

Home Improvement

S22 Table: Effects of initial lockdown and stimulus payments on zip code level home improvement spending

	Dollar Change ($)			Percentage Change (%)		
	All	Republican	Democratic	All	Republican	Democratic
$\beta : \mathbb{1}\{\geq 03-19\}$	-7.635***	-1.894	-15.085***	-3.227**	-0.863	-6.296***
	(1.680)	(1.650)	(3.211)	(1.350)	(1.871)	(1.931)
$\gamma : \mathbb{1}\{\geq 04-11\}$	184.652***	123.582***	261.618***	110.444***	112.940***	107.298***
	(3.111)	(2.641)	(6.056)	(1.862)	(2.568)	(2.691)
Adjusted R^2	0.164	0.113	0.186	0.121	0.122	0.118
N	1464943	824003	640940	1464943	824003	640940

***** 0.01 **0.05 * 0.1**

Note: The regressions include the zip-month fixed effects. The standard errors shown in the parenthesis are clustered at the zip code level.

S23 Table: Effects on home improvement dollar change across geographic regions

	Dependent Variable: Dollar Change ($)							
	New England	Mideast	Southeast	Great Lakes	Plains	Rocky Mountains	Southwest	Far West
$\beta : \mathbb{1}\{\geq 03-19\}$	-2.354	-29.281***	-8.337***	-8.327*	7.352	-2.002	-0.986	-1.619
	(6.222)	(4.708)	(2.746)	(4.663)	(4.598)	(7.575)	(6.302)	(4.189)
$\gamma : \mathbb{1}\{\geq 04-11\}$	126.997***	192.820***	178.781***	235.400***	163.429***	141.030***	220.459***	139.434***
	(10.338)	(9.448)	(5.195)	(10.557)	(9.018)	(10.853)	(8.558)	(6.672)
Adjusted R^2	0.172	0.160	0.148	0.143	0.131	0.091	0.256	0.123
N	64344	201589	449575	223172	117279	46382	185175	178809

***** 0.01 **0.05 * 0.1**

Note: The regressions include the zip-month fixed effects. The standard errors shown in the parenthesis are clustered at the zip code level.

S24 Table: Effects on home improvement percentage change across geographic regions

	Dependent Variable: Percentage Change (%)							
	New England	Mideast	Southeast	Great Lakes	Plains	Rocky Mountains	Southwest	Far West
$\beta : \mathbb{1}\{\geq 03-19\}$	-10.748	-12.069***	-5.540**	3.249	8.373*	-4.031	-2.734	-0.621
	(7.100)	(3.773)	(2.442)	(3.667)	(4.856)	(7.178)	(3.283)	(3.792)
$\gamma : \mathbb{1}\{\geq 04-11\}$	99.472***	108.000***	110.216***	145.284***	130.471***	107.717***	94.980***	77.579***
	(9.710)	(4.857)	(3.272)	(5.105)	(7.116)	(9.628)	(4.902)	(5.104)
Adjusted R^2	0.127	0.122	0.113	0.127	0.128	0.114	0.122	0.111
N	64344	201589	449575	223172	117279	46382	185175	178809

***** 0.01 **0.05 * 0.1**

Note: The regressions include the zip-month fixed effects. The standard errors shown in the parenthesis are clustered at the zip code level.

Home Office

S25 Table: Effects of initial lockdown and stimulus payments on zip code level home office spending

	Dollar Change ($)			Percentage Change (%)		
	All	Republican	Democratic	All	Republican	Democratic
$\beta : \mathbb{1}\{\geq 03-19\}$	5.592***	7.199***	4.792**	31.127***	46.111**	23.670*
	(1.458)	(2.227)	(1.881)	(11.011)	(19.160)	(13.445)
$\gamma : \mathbb{1}\{\geq 04-11\}$	23.172***	20.736***	24.291***	133.344***	136.980***	131.672***
	(1.577)	(2.466)	(2.001)	(14.203)	(24.847)	(17.305)
Adjusted R^2	0.174	0.174	0.174	0.165	0.189	0.153
N	132951	46030	86921	132951	46030	86921

***** 0.01 **0.05 * 0.1**

Note: The regressions include the zip-month fixed effects. The standard errors shown in the parenthesis are clustered at the zip code level.

S26 Table: Effects on home office dollar change across geographic regions

	Dependent Variable: Dollar Change ($)							
	New England	Mideast	Southeast	Great Lakes	Plains	Rocky Mountains	Southwest	Far West
$\beta : \mathbb{1}\{\geq 03-19\}$	0.219	6.990*	4.541*	-2.861	23.516***	10.331	7.149**	5.611
	(4.268)	(3.621)	(2.759)	(5.305)	(7.512)	(6.336)	(2.785)	(4.082)
$\gamma : \mathbb{1}\{\geq 04-11\}$	18.026***	22.564***	27.394***	20.978***	24.279***	15.993	23.709***	17.892***
	(5.915)	(4.056)	(3.097)	(3.794)	(9.196)	(9.884)	(3.841)	(3.420)
Adjusted R^2	0.229	0.142	0.147	0.210	0.212	0.228	0.195	0.213
N	4368	18885	41951	13730	5938	3447	24901	19752

***** 0.01 **0.05 * 0.1**

Note: The regressions include the zip-month fixed effects. The standard errors shown in the parenthesis are clustered at the zip code level.

S27 Table: Effects on home office percentage change across geographic regions

	Dependent Variable: Percentage Change (%)							
	New England	Mideast	Southeast	Great Lakes	Plains	Rocky Mountains	Southwest	Far West
$\beta : \mathbb{1}\{\geq 03-19\}$	-87.450	29.618	29.778	21.545	43.529	13.853	46.024*	47.912*
	(70.257)	(27.751)	(20.743)	(35.842)	(48.114)	(64.531)	(23.568)	(27.301)
$\gamma : \mathbb{1}\{\geq 04-11\}$	88.527	138.524***	154.154***	170.734***	51.199	80.950	123.214***	113.026***
	(62.961)	(33.628)	(24.614)	(44.779)	(76.271)	(86.644)	(35.546)	(39.109)
Adjusted R^2	0.197	0.150	0.156	0.191	0.196	0.203	0.148	0.178
N	4368	18885	41951	13730	5938	3447	24901	19752

***** 0.01 **0.05 * 0.1**

Note: The regressions include the zip-month fixed effects. The standard errors shown in the parenthesis are clustered at the zip code level.

School/Education

S28 Table: Effects of initial lockdown and stimulus payments on zip code level school/education spending

	Dollar Change ($)			Percentage Change (%)		
	All	Republican	Democratic	All	Republican	Democratic
$\beta : \mathbb{1}\{\geq 03-19\}$	-32.268***	-28.065***	-34.929***	-60.632***	-64.735***	-58.036***
	(2.385)	(3.411)	(3.239)	(5.073)	(7.670)	(6.712)
$\gamma : \mathbb{1}\{\geq 04-11\}$	31.249***	25.019***	34.914***	61.353***	63.345***	60.181***
	(2.730)	(4.061)	(3.614)	(6.578)	(10.515)	(8.419)
Adjusted R^2	0.102	0.116	0.095	0.141	0.156	0.131
N	260776	106597	154179	260776	106597	154179

***** 0.01 **0.05 * 0.1**

Note: The regressions include the zip-month fixed effects. The standard errors shown in the parenthesis are clustered at the zip code level.

S29 Table: Effects on school/education dollar change across geographic regions

	Dependent Variable: Dollar Change ($)							
	New England	Mideast	Southeast	Great Lakes	Plains	Rocky Mountains	Southwest	Far West
$\beta : \mathbb{1}\{\geq 03-19\}$	-17.574*	-30.356***	-38.784***	-20.429***	-24.395**	-9.638	-47.597***	-13.242**
	(9.860)	(7.437)	(4.323)	(5.359)	(10.680)	(9.908)	(5.618)	(6.487)
$\gamma : \mathbb{1}\{\geq 04-11\}$	27.225**	16.640*	44.670***	27.694***	31.918***	-5.174	33.471***	19.854***
	(12.605)	(8.844)	(4.810)	(7.392)	(11.013)	(11.985)	(6.571)	(6.939)
Adjusted R^2	0.158	0.110	0.088	0.104	0.108	0.110	0.091	0.137
N	6744	26688	82028	33575	17244	9133	52996	32411

***** 0.01 **0.05 * 0.1**

Note: The regressions include the zip-month fixed effects. The standard errors shown in the parenthesis are clustered at the zip code level.

S30 Table: Effects on school/education percentage change across geographic regions

	Dependent Variable: Percentage Change (%)							
	New England	Mideast	Southeast	Great Lakes	Plains	Rocky Mountains	Southwest	Far West
$\beta : \mathbb{1}\{\geq 03-19\}$	-24.995	-46.464***	-73.428***	-36.041**	-64.295***	-32.553	-86.310***	-28.467*
	(30.382)	(16.440)	(8.694)	(14.605)	(22.458)	(31.724)	(9.506)	(16.962)
$\gamma : \mathbb{1}\{\geq 04-11\}$	90.817**	50.529**	86.373***	71.720***	82.676***	-29.055	41.400***	32.791*
	(41.590)	(20.377)	(11.046)	(19.511)	(25.894)	(44.441)	(13.905)	(19.792)
Adjusted R^2	0.168	0.153	0.130	0.144	0.151	0.140	0.120	0.156
N	6744	26688	82028	33575	17244	9133	52996	32411

***** 0.01 **0.05 * 0.1**

Note: The regressions include the zip-month fixed effects. The standard errors shown in the parenthesis are clustered at the zip code level.

Child Care

S31 Table: Effects of initial lockdown and stimulus payments on zip code level child care spending

	Dollar Change ($)			Percentage Change (%)		
	All	Republican	Democratic	All	Republican	Democratic
$\beta : \mathbb{1}\{\geq 03-19\}$	-73.643***	-43.961***	-93.927***	-49.375***	-31.240***	-61.768***
	(5.631)	(7.762)	(7.619)	(4.463)	(6.545)	(5.950)
$\gamma : \mathbb{1}\{\geq 04-11\}$	-10.844	-14.089	-8.485	-2.570	-1.149	-3.603
	(10.357)	(14.128)	(14.656)	(7.465)	(12.196)	(9.363)
Adjusted R^2	0.166	0.190	0.156	0.205	0.236	0.188
N	41476	15461	26015	41476	15461	26015

***** 0.01 **0.05 * 0.1**

Note: The regressions include the zip-month fixed effects. The standard errors shown in the parenthesis are clustered at the zip code level.

S32 Table: Effects on child care spending change across geographic regions

	Dependent Variable: Percentage Change (%)							
	New England	Mideast	Southeast	Great Lakes	Plains	Rocky Mountains	Southwest	Far West
$\beta : \mathbb{1}\{\geq 03-19\}$	-17.631	-26.120	-76.694***	-67.675***	-77.330**	-37.237	-77.840***	-51.276
	(97.347)	(25.621)	(7.617)	(19.693)	(36.644)	(45.899)	(10.181)	(32.985)
$\gamma : \mathbb{1}\{\geq 04-11\}$	11.090***	46.004	-10.975	85.838**	23.420	-7.123***	-24.760	4.645
	(0.000)	(31.695)	(11.638)	(40.557)	(33.988)	(0.000)	(21.774)	(39.843)
Adjusted R^2	0.342	0.196	0.156	0.241	0.222	0.450	0.154	0.201
N	527	2714	19628	2556	1448	228	13150	1225

***** 0.01 **0.05 * 0.1**

Note: The regressions include the zip-month fixed effects. The standard errors shown in the parenthesis are clustered at the zip code level.

S33 Table: Effects on child care percentage change across geographic regions

	Dependent Variable: Percentage Change (%)							
	New England	Mideast	Southeast	Great Lakes	Plains	Rocky Mountains	Southwest	Far West
$\beta : \mathbb{1}\{\geq 03-19\}$	38.217	-47.777**	-43.779***	-92.860***	-46.869	-159.026	-51.347***	-60.365*
	(73.574)	(19.851)	(6.326)	(24.847)	(32.781)	(102.188)	(7.033)	(32.272)
$\gamma : \mathbb{1}\{\geq 04-11\}$	6.042***	24.798	-8.893	70.947*	50.785	-0.760***	-2.874	-33.897
	(0.000)	(48.427)	(9.027)	(42.610)	(48.181)	(0.000)	(13.744)	(50.865)
Adjusted R^2	0.365	0.229	0.191	0.276	0.254	0.340	0.173	0.225
N	527	2714	19628	2556	1448	228	13150	1225

***** 0.01 **0.05 * 0.1**

Note: The regressions include the zip-month fixed effects. The standard errors shown in the parenthesis are clustered at the zip code level.

.4 Health Spending

Total

S34 Table: Effects of initial lockdown and stimulus payments on zip code level health spending

	Dollar Change ($)			Percentage Change (%)		
	All	Republican	Democratic	All	Republican	Democratic
$\beta : \mathbb{1}\{\geq 03-19\}$	-23.261***	-14.858***	-33.324***	-11.822***	-9.802***	-14.240***
	(1.295)	(1.491)	(2.209)	(0.636)	(0.920)	(0.861)
$\gamma : \mathbb{1}\{\geq 04-11\}$	132.333***	96.419***	173.725***	44.573***	46.656***	42.172***
	(2.223)	(2.130)	(4.053)	(0.824)	(1.197)	(1.113)
Adjusted R^2	0.169	0.120	0.196	0.120	0.120	0.117
N	1471045	800112	670933	1471045	800112	670933

***** 0.01 **0.05 * 0.1**

Note: The regressions include the zip-month fixed effects. The standard errors shown in the parenthesis are clustered at the zip code level.

S35 Table: Effects on health dollar change across geographic region

	Dependent Variable: Dollar Change ($)							
	New England	Mideast	Southeast	Great Lakes	Plains	Rocky Mountains	Southwest	Far West
$\beta : \mathbb{1}\{\geq 03-19\}$	-20.834***	-28.333***	-21.098***	-18.806***	-3.478	3.449	-34.530***	-38.115***
	(4.398)	(3.261)	(2.350)	(3.118)	(3.721)	(6.201)	(4.658)	(3.939)
$\gamma : \mathbb{1}\{\geq 04-11\}$	80.393***	123.298***	133.160***	166.634***	126.917***	104.880***	162.603***	108.321***
	(6.251)	(5.642)	(3.672)	(7.687)	(7.882)	(9.188)	(6.845)	(5.718)
Adjusted R^2	0.196	0.223	0.151	0.250	0.139	0.103	0.146	0.092
N	78606	220289	447491	217978	112783	46492	175051	173444

***** 0.01 **0.05 * 0.1**

Note: The regressions include the zip-month fixed effects. The standard errors shown in the parenthesis are clustered at the zip code level.

S36 Table: Effects on health percentage change across geographic regions

	Dependent Variable: Percentage Change (%)							
	New England	Mideast	Southeast	Great Lakes	Plains	Rocky Mountains	Southwest	Far West
$\beta : \mathbb{1}\{\geq 03-19\}$	-8.164**	-18.510***	-12.026***	-12.928***	-5.080**	0.184	-9.305***	-13.668***
	(3.181)	(1.750)	(1.147)	(1.694)	(2.332)	(3.357)	(1.579)	(1.802)
$\gamma : \mathbb{1}\{\geq 04-11\}$	29.615***	36.430***	50.202***	55.209***	47.226***	39.661***	39.010***	39.802***
	(3.556)	(2.064)	(1.493)	(2.261)	(3.103)	(4.657)	(2.292)	(2.270)
Adjusted R^2	0.131	0.113	0.114	0.135	0.120	0.124	0.119	0.107
N	78606	220289	447491	217978	112783	46492	175051	173444

***** 0.01 **0.05 * 0.1**

Note: The regressions include the zip-month fixed effects. The standard errors shown in the parenthesis are clustered at the zip code level.

Medical Services

S37 Table: Effects of initial lockdown and stimulus payments on zip code level medical services spending

	Dollar Change ($)			Percentage Change (%)		
	All	Republican	Democratic	All	Republican	Democratic
$\beta : \mathbb{1}\{\geq 03-19\}$	-62.229***	-45.439***	-77.830***	-53.095***	-49.804***	-56.153***
	(2.624)	(3.282)	(4.002)	(2.947)	(4.261)	(4.078)
$\gamma : \mathbb{1}\{\geq 04-11\}$	53.355***	45.595***	60.342***	56.999***	55.610***	58.250***
	(2.830)	(3.518)	(4.338)	(3.761)	(5.429)	(5.215)
Adjusted R^2	0.101	0.093	0.106	0.127	0.136	0.119
N	342838	164579	178259	342838	164579	178259

***** 0.01 **0.05 * 0.1**

Note: The regressions include the zip-month fixed effects. The standard errors shown in the parenthesis are clustered at the zip code level.

S38 Table: Health medical services spending difference by geographic regions

	Dependent Variable: Dollar Change ($)							
	New England	Mideast	Southeast	Great Lakes	Plains	Rocky Mountains	Southwest	Far West
$\beta : \mathbb{1}\{\geq 03-19\}$	-45.486***	-49.971***	-62.532***	-53.329***	-48.465***	-49.796***	-87.467***	-40.838***
	(12.186)	(7.004)	(4.166)	(6.714)	(8.404)	(10.769)	(7.216)	(7.196)
$\gamma : \mathbb{1}\{\geq 04-11\}$	42.239***	39.343***	60.936***	50.424***	53.893***	62.789***	52.556***	43.490***
	(13.698)	(7.345)	(4.554)	(7.875)	(11.407)	(13.079)	(7.357)	(9.019)
Adjusted R^2	0.150	0.098	0.083	0.094	0.088	0.099	0.118	0.120
N	6622	34109	119895	40898	21734	12589	71166	35864

***** 0.01 **0.05 * 0.1**

Note: The regressions include the zip-month fixed effects. The standard errors shown in the parenthesis are clustered at the zip code level.

S39 Table: Health medical services percentage difference by geographic regions

	Dependent Variable: Percentage Change (%)							
	New England	Mideast	Southeast	Great Lakes	Plains	Rocky Mountains	Southwest	Far West
$\beta : \mathbb{1}\{\geq 03-19\}$	-46.095*	-63.773***	-54.133***	-60.250***	-32.981***	-44.992***	-56.194***	-41.508***
	(26.495)	(9.975)	(5.017)	(9.243)	(12.308)	(15.687)	(5.546)	(9.603)
$\gamma : \mathbb{1}\{\geq 04-11\}$	79.043***	58.867***	60.212***	70.599***	62.848***	69.702***	45.641***	38.755***
	(29.434)	(11.338)	(6.441)	(11.012)	(13.119)	(18.873)	(8.099)	(12.601)
Adjusted R^2	0.196	0.138	0.120	0.133	0.132	0.120	0.108	0.139
N	6622	34109	119895	40898	21734	12589	71166	35864

***** 0.01 **0.05 * 0.1**

Note: The regressions include the zip-month fixed effects. The standard errors shown in the parenthesis are clustered at the zip code level.

Drug Stores

S40 Table: Effects of initial lockdown and stimulus payments on zip code level drug stores spending

	Dollar Change ($)			Percentage Change (%)		
	All	Republican	Democratic	All	Republican	Democratic
$\beta : \mathbb{1}\{\geq 03-19\}$	-9.908***	-3.518***	-15.499***	-8.224***	-6.394***	-9.825***
	(0.646)	(0.686)	(1.045)	(0.806)	(1.245)	(1.048)
$\gamma : \mathbb{1}\{\geq 04-11\}$	38.363***	23.730***	50.736***	31.205***	28.508***	33.486***
	(0.936)	(0.936)	(1.513)	(1.050)	(1.639)	(1.354)
Adjusted R^2	0.150	0.117	0.162	0.122	0.127	0.115
N	1010485	474365	536120	1010485	474365	536120

***** 0.01 **0.05 * 0.1**

Note: The regressions include the zip-month fixed effects. The standard errors shown in the parenthesis are clustered at the zip code level.

S41 Table: Effects on drug stores dollar change across geographic regions

	Dependent Variable: Dollar Change ($)							
	New England	Mideast	Southeast	Great Lakes	Plains	Rocky Mountains	Southwest	Far West
$\beta : \mathbb{1}\{\geq 03-19\}$	-15.866***	-9.460***	-10.874***	-15.433***	-8.283***	-3.434	-8.353***	-3.332**
	(2.700)	(1.687)	(1.066)	(2.219)	(2.377)	(2.779)	(1.816)	(1.497)
$\gamma : \mathbb{1}\{\geq 04-11\}$	37.247***	46.269***	27.176***	57.974***	39.307***	25.438***	40.568***	33.359***
	(3.931)	(2.670)	(1.374)	(3.188)	(3.739)	(3.895)	(2.579)	(2.225)
Adjusted R^2	0.137	0.172	0.115	0.177	0.113	0.080	0.184	0.121
N	51366	147260	308623	149207	64087	26302	128362	135491

***** 0.01 **0.05 * 0.1**

Note: The regressions include the zip-month fixed effects. The standard errors shown in the parenthesis are clustered at the zip code level.

S42 Table: Effects on drug stores percentage change across geographic regions

	Dependent Variable: Percentage Change (%)							
	New England	Mideast	Southeast	Great Lakes	Plains	Rocky Mountains	Southwest	Far West
$\beta : \mathbb{1}\{\geq 03-19\}$	-19.457***	-8.285***	-9.011***	-9.391***	-9.020***	-6.938	-3.748*	-5.076**
	(3.875)	(2.111)	(1.431)	(2.207)	(3.421)	(5.706)	(1.930)	(2.251)
$\gamma : \mathbb{1}\{\geq 04-11\}$	39.985***	30.797***	24.757***	40.509***	31.968***	40.565***	29.301***	32.110***
	(4.534)	(2.714)	(1.853)	(2.963)	(4.344)	(7.514)	(2.774)	(2.753)
Adjusted R^2	0.134	0.122	0.120	0.136	0.114	0.097	0.123	0.098
N	51366	147260	308623	149207	64087	26302	128362	135491

***** 0.01 **0.05 * 0.1**

Note: The regressions include the zip-month fixed effects. The standard errors shown in the parenthesis are clustered at the zip code level.

Liquor Stores and Cigar Stores

S43 Table: Effects of initial lockdown and stimulus payments on zip code level liquor stores and cigar stores spending

	Dollar Change ($)			Percentage Change (%)		
	All	Republican	Democratic	All	Republican	Democratic
$\beta : \mathbb{1}\{\geq 03-19\}$	35.067***	22.621***	48.155***	18.136***	18.476***	17.778***
	(0.924)	(0.803)	(1.676)	(0.728)	(1.040)	(1.016)
$\gamma : \mathbb{1}\{\geq 04-11\}$	44.528***	27.378***	61.805***	31.996***	29.667***	34.341***
	(1.134)	(0.919)	(2.050)	(0.904)	(1.298)	(1.257)
Adjusted R^2	0.352	0.244	0.379	0.132	0.131	0.134
N	1096502	560205	536297	1096502	560205	536297

***** 0.01 **0.05 * 0.1**

Note: The regressions include the zip-month fixed effects. The standard errors shown in the parenthesis are clustered at the zip code level.

S44 Table: Effects on liquor stores and cigar stores dollar change across geographic regions

	Dependent Variable: Dollar Change ($)							
	New England	Mideast	Southeast	Great Lakes	Plains	Rocky Mountains	Southwest	Far West
$\beta : \mathbb{1}\{\geq 03-19\}$	26.583***	27.531***	44.642***	35.921***	28.171***	42.636***	54.859***	3.677*
	(2.547)	(3.117)	(1.629)	(2.842)	(2.076)	(4.071)	(2.428)	(1.942)
$\gamma : \mathbb{1}\{\geq 04-11\}$	30.851***	57.852***	36.965***	72.683***	35.298***	17.851***	34.492***	39.126***
	(3.184)	(3.485)	(1.535)	(4.693)	(2.883)	(4.059)	(2.399)	(2.555)
Adjusted R^2	0.384	0.419	0.356	0.400	0.277	0.211	0.294	0.136
N	58134	167013	323255	161071	84868	38599	133171	131179

***** 0.01 **0.05 * 0.1**

Note: The regressions include the zip-month fixed effects. The standard errors shown in the parenthesis are clustered at the zip code level.

S45 Table: Effects on liquor stores and cigar stores percentage change across geographic regions

	Dependent Variable: Percentage Change (%)							
	New England	Mideast	Southeast	Great Lakes	Plains	Rocky Mountains	Southwest	Far West
$\beta : \mathbb{1}\{\geq 03-19\}$	16.621***	5.209***	29.551***	9.146***	16.213***	17.919***	33.206***	2.527
	(2.946)	(1.980)	(1.338)	(1.847)	(2.413)	(3.393)	(2.063)	(2.068)
$\gamma : \mathbb{1}\{\geq 04-11\}$	27.048***	33.830***	31.393***	40.431***	32.031***	5.898	28.384***	33.031***
	(3.706)	(2.187)	(1.713)	(2.375)	(3.191)	(4.449)	(2.692)	(2.597)
Adjusted R^2	0.149	0.130	0.126	0.151	0.143	0.141	0.122	0.119
N	58134	167013	323255	161071	84868	38599	133171	131179

***** 0.01 **0.05 * 0.1**

Note: The regressions include the zip-month fixed effects. The standard errors shown in the parenthesis are clustered at the zip code level.

.5 General Purpose Merchandise Spending

Total

S46 Table: Effects of initial lockdown and stimulus payments on zip code level general purpose merchandise spending

	Dollar Change ($)			Percentage Change (%)		
	All	Republican	Democratic	All	Republican	Democratic
$\beta : \mathbb{1}\{\geq 03-19\}$	-36.786***	-22.770***	-60.344***	-8.094***	-7.184***	-9.624***
	(1.730)	(1.516)	(3.856)	(0.622)	(0.810)	(0.963)
$\gamma : \mathbb{1}\{\geq 04-11\}$	296.691***	177.487***	490.613***	67.158***	62.829***	74.201***
	(5.112)	(3.330)	(11.849)	(0.869)	(1.117)	(1.375)
Adjusted R^2	0.277	0.234	0.294	0.140	0.139	0.140
N	1803147	1122319	680828	1803147	1122319	680828

***** 0.01 **0.05 * 0.1**

Note: The regressions include the zip-month fixed effects. The standard errors shown in the parenthesis are clustered at the zip code level.

S47 Table: Effects on general purpose merchandise dollar change across geographic regions

	Dependent Variable: Dollar Change ($)							
	New England	Mideast	Southeast	Great Lakes	Plains	Rocky Mountains	Southwest	Far West
$\beta : \mathbb{1}\{\geq 03-19\}$	-2.147	-8.678*	-38.535***	-18.518***	-10.467**	-46.693***	-77.077***	-79.913***
	(8.180)	(4.753)	(2.714)	(4.347)	(5.193)	(7.653)	(5.111)	(7.292)
$\gamma : \mathbb{1}\{\geq 04-11\}$	290.519***	369.306***	264.699***	405.927***	211.330***	136.249***	245.401***	292.834***
	(22.495)	(17.469)	(7.080)	(18.301)	(13.771)	(10.542)	(9.410)	(13.516)
Adjusted R^2	0.249	0.332	0.287	0.354	0.229	0.114	0.229	0.125
N	74216	242200	595244	284845	155468	49646	222935	179817

***** 0.01 **0.05 * 0.1**

Note: The regressions include the zip-month fixed effects. The standard errors shown in the parenthesis are clustered at the zip code level.

S48 Table: Effects on general purpose merchandise percentage change across geographic regions

	Dependent Variable: Percentage Change (%)							
	New England	Mideast	Southeast	Great Lakes	Plains	Rocky Mountains	Southwest	Far West
$\beta : \mathbb{1}\{\geq 03-19\}$	-1.049	-3.498*	-8.186***	-7.638***	-1.412	-23.436***	-10.775***	-15.557***
	(3.853)	(1.856)	(0.998)	(1.711)	(2.162)	(4.192)	(1.440)	(2.011)
$\gamma : \mathbb{1}\{\geq 04-11\}$	79.350***	76.739***	64.445***	84.161***	58.582***	53.813***	46.338***	63.550***
	(4.759)	(2.419)	(1.368)	(2.427)	(3.229)	(5.225)	(2.044)	(2.843)
Adjusted R^2	0.139	0.129	0.139	0.149	0.122	0.112	0.143	0.116
N	74216	242200	595244	284845	155468	49646	222935	179817

***** 0.01 **0.05 * 0.1**

Note: The regressions include the zip-month fixed effects. The standard errors shown in the parenthesis are clustered at the zip code level.

Wholesale Clubs

S49 Table: Effects of initial lockdown and stimulus payments on zip code level wholesale clubs spending

	Dollar Change ($)			Percentage Change (%)		
	All	Republican	Democratic	All	Republican	Democratic
$\beta : \mathbb{1}\{\geq 03-19\}$	26.638***	19.629***	30.938***	51.534***	70.515***	39.891***
	(2.331)	(3.652)	(3.018)	(7.170)	(11.851)	(8.988)
$\gamma : \mathbb{1}\{\geq 04-11\}$	73.307***	51.506***	86.007***	125.289***	109.802***	134.310***
	(3.393)	(4.666)	(4.600)	(9.859)	(16.720)	(12.183)
Adjusted R^2	0.189	0.171	0.196	0.131	0.141	0.125
N	285230	108211	177019	285230	108211	177019

***** 0.01 **0.05 * 0.1**

Note: The regressions include the zip-month fixed effects. The standard errors shown in the parenthesis are clustered at the zip code level.

S50 Table: Effects on wholesale clubs dollar change across geographic regions

	Dependent Variable: Spending Change ($)							
	New England	Mideast	Southeast	Great Lakes	Plains	Rocky Mountains	Southwest	Far West
$\beta : \mathbb{1}\{\geq 03-19\}$	13.433	42.014***	28.177***	26.644***	30.351***	14.677	23.408***	20.096***
	(10.967)	(6.943)	(4.305)	(6.855)	(8.863)	(12.448)	(5.496)	(5.622)
$\gamma : \mathbb{1}\{\geq 04-11\}$	74.622***	78.795***	60.630***	130.347***	67.851***	58.032***	60.628***	71.159***
	(13.279)	(8.759)	(6.171)	(14.369)	(9.752)	(14.279)	(7.124)	(8.270)
Adjusted R^2	0.213	0.178	0.184	0.173	0.180	0.127	0.249	0.154
N	6212	33107	81380	32551	19658	11087	56683	44702

***** 0.01 **0.05 * 0.1**

Note: The regressions include the zip-month fixed effects. The standard errors shown in the parenthesis are clustered at the zip code level.

S51 Table: Effects on wholesale clubs percentage change across geographic regions

	Dependent Variable: Percentage Change (%)							
	New England	Mideast	Southeast	Great Lakes	Plains	Rocky Mountains	Southwest	Far West
$\beta : \mathbb{1}\{\geq 03-19\}$	29.257	68.326***	63.236***	44.412**	62.552**	24.569	36.654**	47.074**
	(46.855)	(21.034)	(12.938)	(22.017)	(28.392)	(41.837)	(15.018)	(19.462)
$\gamma : \mathbb{1}\{\geq 04-11\}$	178.867***	136.090***	120.229***	192.796***	96.617**	86.108	78.882***	142.038***
	(46.520)	(26.671)	(19.131)	(31.280)	(39.607)	(56.779)	(19.678)	(25.487)
Adjusted R^2	0.172	0.143	0.133	0.139	0.132	0.121	0.114	0.121
N	6212	33107	81380	32551	19658	11087	56683	44702

***** 0.01 **0.05 * 0.1**

Note: The regressions include the zip-month fixed effects. The standard errors shown in the parenthesis are clustered at the zip code level.

Discount Stores

S52 Table: Effects of initial lockdown and stimulus payments on zip code level discount stores spending

	Dollar Change ($)			Percentage Change (%)		
	All	Republican	Democratic	All	Republican	Democratic
$\beta : \mathbb{1}\{\geq 03-19\}$	-13.181***	-7.110***	-23.723***	-4.755***	-4.162***	-5.785***
	(1.535)	(1.244)	(3.598)	(0.791)	(1.001)	(1.290)
$\gamma : \mathbb{1}\{\geq 04-11\}$	203.109***	121.011***	341.540***	79.296***	68.249***	97.924***
	(3.876)	(2.487)	(9.159)	(1.147)	(1.413)	(1.928)
Adjusted R^2	0.193	0.174	0.202	0.136	0.136	0.136
N	1476475	929384	547091	1476475	929384	547091

***** 0.01 **0.05 * 0.1**

Note: The regressions include the zip-month fixed effects. The standard errors shown in the parenthesis are clustered at the zip code level.

S53 Table: Effects on discount stores dollar change across geographic regions

	Dependent Variable: Dollar Change ($)							
	New England	Mideast	Southeast	Great Lakes	Plains	Rocky Mountains	Southwest	Far West
$\beta : \mathbb{1}\{\geq 03-19\}$	21.569**	16.170***	-15.025***	14.241***	2.944	-34.018***	-47.826***	-62.900***
	(10.385)	(4.798)	(1.927)	(4.230)	(5.501)	(7.084)	(3.823)	(7.322)
$\gamma : \mathbb{1}\{\geq 04-11\}$	282.466***	282.419***	162.400***	272.062***	147.716***	101.463***	154.720***	228.226***
	(24.385)	(14.989)	(4.584)	(13.218)	(11.011)	(9.249)	(6.310)	(12.697)
Adjusted R^2	0.184	0.259	0.191	0.233	0.142	0.088	0.192	0.100
N	50301	183572	532169	225055	123383	28625	194812	139004

***** 0.01 **0.05 * 0.1**

Note: The regressions include the zip-month fixed effects. The standard errors shown in the parenthesis are clustered at the zip code level.

S54 Table: Effects on discount stores percentage change across geographic regions

	Dependent Variable: Percentage Change (%)							
	New England	Mideast	Southeast	Great Lakes	Plains	Rocky Mountains	Southwest	Far West
$\beta : \mathbb{1}\{\geq 03-19\}$	3.814	6.514***	-6.286***	1.174	4.097	-38.123***	-13.170***	-15.129***
	(5.157)	(2.469)	(1.214)	(2.249)	(2.804)	(6.305)	(1.797)	(2.715)
$\gamma : \mathbb{1}\{\geq 04-11\}$	103.096***	96.729***	72.006***	105.737***	69.169***	69.461***	51.499***	75.015***
	(6.732)	(3.400)	(1.698)	(3.448)	(4.030)	(9.275)	(2.677)	(3.728)
Adjusted R^2	0.148	0.131	0.131	0.145	0.123	0.098	0.130	0.110
N	50301	183572	532169	225055	123383	28625	194812	139004

***** 0.01 **0.05 * 0.1**

Note: The regressions include the zip-month fixed effects. The standard errors shown in the parenthesis are clustered at the zip code level.

Department Stores

S55 Table: Effects of initial lockdown and stimulus payments on zip code level department stores spending

	Dollar Change ($)			Percentage Change (%)		
	All	Republican	Democratic	All	Republican	Democratic
$\beta : \mathbb{1}\{\geq 03-19\}$	-36.047***	-25.160***	-46.437***	-44.908***	-46.349***	-43.534***
	(1.283)	(1.341)	(2.137)	(2.070)	(3.091)	(2.767)
$\gamma : \mathbb{1}\{\geq 04-11\}$	73.054***	48.362***	95.353***	101.423***	99.653***	103.021***
	(1.789)	(1.586)	(3.017)	(2.816)	(4.207)	(3.778)
Adjusted R^2	0.121	0.104	0.129	0.116	0.122	0.110
N	582843	284687	298156	582843	284687	298156

***** 0.01 **0.05 * 0.1**

Note: The regressions include the zip-month fixed effects. The standard errors shown in the parenthesis are clustered at the zip code level.

S56 Table: Effects on department stores dollar change across geographic regions

	Dependent Variable: Spending Change ($)							
	New England	Mideast	Southeast	Great Lakes	Plains	Rocky Mountains	Southwest	Far West
$\beta : \mathbb{1}\{\geq 03-19\}$	-25.884***	-36.972***	-35.334***	-45.273***	-41.110***	-23.336***	-40.618***	-22.946***
	(6.401)	(4.172)	(2.098)	(3.961)	(4.293)	(4.675)	(3.448)	(3.082)
$\gamma : \mathbb{1}\{\geq 04-11\}$	63.288***	115.386***	61.717***	116.877***	72.004***	28.157***	46.661***	49.654***
	(6.774)	(6.791)	(2.409)	(6.234)	(5.857)	(5.585)	(3.800)	(3.673)
Adjusted R^2	0.127	0.122	0.104	0.117	0.101	0.094	0.167	0.098
N	17257	71616	183800	84764	40234	17705	92389	75221

***** 0.01 **0.05 * 0.1**

Note: The regressions include the zip-month fixed effects. The standard errors shown in the parenthesis are clustered at the zip code level.

S57 Table: Effects on department stores percentage change across geographic regions

	Dependent Variable: Percentage Change (%)							
	New England	Mideast	Southeast	Great Lakes	Plains	Rocky Mountains	Southwest	Far West
$\beta : \mathbb{1}\{\geq 03-19\}$	-50.088***	-34.206***	-45.005***	-49.801***	-52.914***	-48.218***	-48.448***	-38.034***
	(12.223)	(5.797)	(3.756)	(5.380)	(8.239)	(11.166)	(4.640)	(6.264)
$\gamma : \mathbb{1}\{\geq 04-11\}$	121.116***	123.227***	101.639***	143.735***	93.868***	52.797***	75.228***	69.942***
	(15.853)	(7.266)	(4.993)	(7.718)	(10.494)	(17.896)	(6.577)	(8.228)
Adjusted R^2	0.139	0.128	0.111	0.124	0.118	0.112	0.109	0.106
N	17257	71616	183800	84764	40234	17705	92389	75221

***** 0.01 **0.05 * 0.1**

Note: The regressions include the zip-month fixed effects. The standard errors shown in the parenthesis are clustered at the zip code level.

.6 Food Spending

Total

S58 Table: Effects of initial lockdown and stimulus payments on zip code level food spending

	Dollar Change ($)			Percentage Change (%)		
	All	Republican	Democratic	All	Republican	Democratic
$\beta : \mathbb{1}\{\geq 03-19\}$	-414.792***	-265.317***	-663.623***	-21.651***	-19.074***	-25.941***
	(5.971)	(5.578)	(12.418)	(0.286)	(0.389)	(0.397)
$\gamma : \mathbb{1}\{\geq 04-11\}$	813.651***	620.880***	1122.352***	38.729***	42.836***	32.152***
	(11.677)	(10.881)	(24.529)	(0.376)	(0.514)	(0.520)
Adjusted R^2	0.249	0.193	0.287	0.162	0.156	0.175
N	2250916	1399538	851378	2250916	1399538	851378

***** 0.01 **0.05 * 0.1**

Note: The regressions include the zip-month fixed effects. The standard errors shown in the parenthesis are clustered at the zip code level.

S59 Table: Effects on food dollar change across geographic regions

	Dependent Variable: Dollar Change ($)							
	New England	Mideast	Southeast	Great Lakes	Plains	Rocky Mountains	Southwest	Far West
$\beta : \mathbb{1}\{\geq 03-19\}$	-215.851***	-228.818***	-464.087***	-334.420***	-204.830***	-480.004***	-747.475***	-554.682***
	(11.281)	(8.744)	(12.677)	(12.279)	(10.969)	(31.936)	(26.313)	(16.304)
$\gamma : \mathbb{1}\{\geq 04-11\}$	327.764***	464.208***	1034.103***	988.375***	599.097***	606.805***	1140.926***	649.679***
	(22.486)	(20.538)	(24.348)	(37.494)	(29.400)	(42.005)	(38.011)	(27.531)
Adjusted R^2	0.227	0.378	0.192	0.337	0.209	0.117	0.265	0.220
N	122062	332401	667516	367148	207829	71851	249687	235181

***** 0.01 **0.05 * 0.1**

Note: The regressions include the zip-month fixed effects. The standard errors shown in the parenthesis are clustered at the zip code level.

S60 Table: Effects on food-related percentage change across geographic regions

	Dependent Variable: Percentage Change (%)							
	New England	Mideast	Southeast	Great Lakes	Plains	Rocky Mountains	Southwest	Far West
$\beta : \mathbb{1}\{\geq 03-19\}$	-30.436***	-23.233***	-19.974***	-22.646***	-17.267***	-23.040***	-17.918***	-25.638***
	(1.416)	(0.786)	(0.512)	(0.772)	(1.030)	(1.481)	(0.676)	(0.769)
$\gamma : \mathbb{1}\{\geq 04-11\}$	27.601***	29.637***	47.051***	50.050***	38.058***	28.549***	34.039***	25.855***
	(1.551)	(0.926)	(0.678)	(1.029)	(1.457)	(2.277)	(0.925)	(0.933)
Adjusted R^2	0.148	0.142	0.155	0.182	0.139	0.151	0.158	0.179
N	122062	332401	667516	367148	207829	71851	249687	235181

***** 0.01 **0.05 * 0.1**

Note: The regressions include the zip-month fixed effects. The standard errors shown in the parenthesis are clustered at the zip code level.

Restaurants

S61 Table: Effects of initial lockdown and stimulus payments on zip code level restaurants spending

	Dollar Change ($)			Percentage Change (%)		
	All	Republican	Democratic	All	Republican	Democratic
$\beta : \mathbb{1}\{\geq 03-19\}$	-375.228***	-229.036***	-610.941***	-42.387***	-38.985***	-47.872***
	(4.097)	(3.303)	(8.613)	(0.248)	(0.336)	(0.346)
$\gamma : \mathbb{1}\{\geq 04-11\}$	211.148***	142.876***	317.706***	26.040***	27.794***	23.303***
	(3.162)	(2.446)	(6.968)	(0.279)	(0.391)	(0.372)
Adjusted R^2	0.395	0.352	0.428	0.198	0.184	0.229
N	2145451	1318079	827372	2145451	1318079	827372

***** 0.01 **0.05 * 0.1**

Note: The regressions include the zip-month fixed effects. The standard errors shown in the parenthesis are clustered at the zip code level.

S62 Table: Effects on restaurants dollar change across geographic regions

	Dependent Variable: Dollar Change ($)							
	New England	Mideast	Southeast	Great Lakes	Plains	Rocky Mountains	Southwest	Far West
$\beta : \mathbb{1}\{\geq 03-19\}$	-238.571***	-326.747***	-383.259***	-340.413***	-220.881***	-353.096***	-528.180***	-505.073***
	(10.848)	(10.594)	(7.840)	(10.527)	(9.229)	(17.898)	(14.285)	(11.562)
$\gamma : \mathbb{1}\{\geq 04-11\}$	132.075***	222.262***	235.010***	249.478***	176.198***	81.491***	236.011***	147.530***
	(8.726)	(10.145)	(5.620)	(9.685)	(9.604)	(7.651)	(7.874)	(5.788)
Adjusted R^2	0.313	0.376	0.355	0.468	0.297	0.330	0.424	0.406
N	113791	311442	648672	349024	190904	66147	242005	225598

***** 0.01 **0.05 * 0.1**

Note: The regressions include the zip-month fixed effects. The standard errors shown in the parenthesis are clustered at the zip code level.

S63 Table: Effects on restaurants percentage change across geographic regions

	Dependent Variable: Percentage Change (%)							
	New England	Mideast	Southeast	Great Lakes	Plains	Rocky Mountains	Southwest	Far West
$\beta : \mathbb{1}\{\geq 03-19\}$	-52.051***	-46.805***	-40.720***	-44.602***	-37.195***	-44.932***	-33.823***	-46.396***
	(1.280)	(0.718)	(0.426)	(0.666)	(0.893)	(1.455)	(0.559)	(0.658)
$\gamma : \mathbb{1}\{\geq 04-11\}$	21.788***	26.290***	30.014***	30.717***	27.549***	12.118***	20.755***	17.117***
	(1.266)	(0.756)	(0.474)	(0.781)	(1.099)	(1.681)	(0.702)	(0.655)
Adjusted R^2	0.176	0.174	0.200	0.204	0.162	0.187	0.195	0.233
N	113791	311442	648672	349024	190904	66147	242005	225598

***** 0.01 **0.05 * 0.1**

Note: The regressions include the zip-month fixed effects. The standard errors shown in the parenthesis are clustered at the zip code level.

Grocery

S64 Table: Effects of initial lockdown and stimulus payments on zip code level grocery spending

	Dollar Change ($)			Percentage Change (%)		
	All	Republican	Democratic	All	Republican	Democratic
$\beta : \mathbb{1}\{\geq 03-19\}$	-62.824***	-55.841***	-73.974***	-7.053***	-7.623***	-6.142***
	(4.193)	(4.226)	(8.546)	(0.476)	(0.646)	(0.680)
$\gamma : \mathbb{1}\{\geq 04-11\}$	665.329***	534.676***	865.998***	54.371***	60.180***	45.449***
	(9.968)	(9.822)	(20.107)	(0.655)	(0.885)	(0.945)
Adjusted R^2	0.216	0.163	0.251	0.139	0.136	0.145
N	2059416	1260863	798553	2059416	1260863	798553

***** 0.01 **0.05 * 0.1**

Note: The regressions include the zip-month fixed effects. The standard errors shown in the parenthesis are clustered at the zip code level.

S65 Table: Effects on grocery dollar change across geographic regions

	Dependent Variable: Dollar Change ($)							
	New England	Mideast	Southeast	Great Lakes	Plains	Rocky Mountains	Southwest	Far West
$\beta : \mathbb{1}\{\geq 03-19\}$	13.092	89.520***	-99.726***	-12.647	-3.326	-173.912***	-247.960***	-73.808***
	(9.105)	(8.335)	(8.215)	(10.193)	(10.510)	(20.443)	(17.124)	(9.833)
$\gamma : \mathbb{1}\{\geq 04-11\}$	230.743***	285.615***	857.412***	834.051***	494.061***	574.768***	952.408***	535.358***
	(17.564)	(13.461)	(20.911)	(32.050)	(24.542)	(39.432)	(33.336)	(24.308)
Adjusted R^2	0.195	0.320	0.167	0.276	0.185	0.103	0.257	0.181
N	105717	296317	623611	327075	182889	65955	237772	222697

***** 0.01 **0.05 * 0.1**

Note: The regressions include the zip-month fixed effects. The standard errors shown in the parenthesis are clustered at the zip code level.

S66 Table: Effects on grocery percentage change across geographic regions

	Dependent Variable: Percentage Change (%)							
	New England	Mideast	Southeast	Great Lakes	Plains	Rocky Mountains	Southwest	Far West
$\beta : \mathbb{1}\{\geq 03-19\}$	-7.792***	0.483	-8.666***	-8.425***	-3.902**	-10.927***	-9.842***	-7.811***
	(2.488)	(1.378)	(0.847)	(1.272)	(1.702)	(2.446)	(1.107)	(1.299)
$\gamma : \mathbb{1}\{\geq 04-11\}$	37.938***	35.235***	66.371***	72.405***	53.670***	45.623***	48.728***	39.223***
	(2.942)	(1.717)	(1.158)	(1.783)	(2.394)	(3.701)	(1.655)	(1.713)
Adjusted R^2	0.122	0.120	0.131	0.153	0.134	0.146	0.144	0.140
N	105717	296317	623611	327075	182889	65955	237772	222697

***** 0.01 **0.05 * 0.1**

Note: The regressions include the zip-month fixed effects. The standard errors shown in the parenthesis are clustered at the zip code level.

.7 Entertainment Spending

Total

S67 Table: Effects of initial lockdown and stimulus payments on zip code level entertainment spending

	Dollar Change ($)			Percentage Change (%)		
	All	Republican	Democratic	All	Republican	Democratic
$\beta : \mathbb{1}\{\geq 03-19\}$	-109.533***	-72.870***	-161.390***	-32.198***	-28.593***	-37.296***
	(1.843)	(1.759)	(3.598)	(0.718)	(1.010)	(0.981)
$\gamma : \mathbb{1}\{\geq 04-11\}$	234.709***	166.371***	327.795***	72.069***	76.958***	65.409***
	(3.442)	(2.979)	(6.893)	(0.974)	(1.368)	(1.345)
Adjusted R^2	0.288	0.191	0.336	0.134	0.130	0.138
N	1706646	992200	714446	1706646	992200	714446

***** 0.01 **0.05 * 0.1**

Note: The regressions include the zip-month fixed effects. The standard errors shown in the parenthesis are clustered at the zip code level.

S68 Table: Effects on entertainment dollar change across geographic regions

	Dependent Variable: Dollar Change ($)							
	New England	Mideast	Southeast	Great Lakes	Plains	Rocky Mountain	Southwest	Far West
$\beta : \mathbb{1}\{\geq 03-19\}$	-66.682***	-113.455***	-120.933***	-100.217***	-63.047***	-80.604***	-122.067***	-133.735***
	(5.562)	(5.472)	(3.412)	(4.356)	(4.366)	(8.401)	(5.198)	(6.592)
$\gamma : \mathbb{1}\{\geq 04-11\}$	164.402***	283.152***	227.726***	307.628***	193.065***	152.557***	233.514***	172.602***
	(10.646)	(11.566)	(5.636)	(10.927)	(10.082)	(12.165)	(7.950)	(7.292)
Adjusted R^2	0.251	0.440	0.204	0.316	0.236	0.196	0.275	0.191
N	83764	246437	507455	266826	143960	54664	204676	200585

***** 0.01 **0.05 * 0.1**

Note: The regressions include the zip-month fixed effects. The standard errors shown in the parenthesis are clustered at the zip code level.

S69 Table: Effects on entertainment percentage change across geographic regions

	Dependent Variable: Percentage Change (%)							
	New England	Mideast	Southeast	Great Lakes	Plains	Rocky Mountains	Southwest	Far West
$\beta : \mathbb{1}\{\geq 03-19\}$	-37.682***	-31.202***	-38.529***	-27.429***	-21.652***	-27.285***	-24.415***	-38.722***
	(3.566)	(2.110)	(1.285)	(1.902)	(2.608)	(4.165)	(1.683)	(1.927)
$\gamma : \mathbb{1}\{\geq 04-11\}$	70.043***	74.203***	75.035***	98.654***	79.044***	49.461***	56.286***	43.291***
	(4.665)	(2.643)	(1.750)	(2.689)	(3.577)	(5.069)	(2.396)	(2.357)
Adjusted R^2	0.127	0.141	0.126	0.135	0.123	0.129	0.136	0.126
N	83764	246437	507455	266826	143960	54664	204676	200585

***** 0.01 **0.05 * 0.1**

Note: The regressions include the zip-month fixed effects. The standard errors shown in the parenthesis are clustered at the zip code level.

Outdoor Recreational Activities

S70 Table: Effects of initial lockdown and stimulus payments on zip code level outdoor recreational activities spending

	Spending Change ($)			Percentage Change (%)		
	All	Republican	Democratic	All	Republican	Democratic
$\beta : \mathbb{1}\{\geq 03-19\}$	-110.800***	-68.879***	-156.947***	-88.356***	-80.770***	-96.707***
	(1.906)	(1.705)	(3.395)	(1.042)	(1.556)	(1.355)
$\gamma : \mathbb{1}\{\geq 04-11\}$	11.456***	13.057***	9.775***	12.729***	18.763***	6.393***
	(1.187)	(1.384)	(1.952)	(1.273)	(1.970)	(1.585)
Adjusted R^2	0.374	0.127	0.440	0.134	0.133	0.133
N	957669	491093	466576	957669	491093	466576

***** 0.01 **0.05 * 0.1**

Note: The regressions include the zip-month fixed effects. The standard errors shown in the parenthesis are clustered at the zip code level.

S71 Table: Effects on outdoor recreational activities dollar change across geographic regions

	Dependent Variable: Dollar Change ($)							
	New England	Mideast	Southeast	Great Lakes	Plains	Rocky Mountain	Southwest	Far West
$\beta : \mathbb{1}\{\geq 03-19\}$	-71.767***	-127.373***	-103.404***	-107.291***	-74.073***	-103.851***	-118.763***	-140.293***
	(5.714)	(6.062)	(3.044)	(4.809)	(4.990)	(8.692)	(5.709)	(6.161)
$\gamma : \mathbb{1}\{\geq 04-11\}$	8.573	1.784	14.540***	20.597***	19.470***	11.688*	10.044***	1.955
	(5.312)	(3.771)	(1.937)	(2.878)	(4.258)	(6.249)	(3.077)	(3.774)
Adjusted R^2	0.144	0.659	0.159	0.146	0.141	0.163	0.236	0.239
N	35199	112774	290455	135802	71776	33360	144000	134970

***** 0.01 **0.05 * 0.1**

Note: The regressions include the zip-month fixed effects. The standard errors shown in the parenthesis are clustered at the zip code level.

S72 Table: Effects on outdoor recreational activities percentage change across geographic regions

	Dependent Variable: Percentage Change (%)							
	New England	Mideast	Southeast	Great Lakes	Plains	Rocky Mountain	Southwest	Far West
$\beta : \mathbb{1}\{\geq 03-19\}$	-90.217***	-94.560***	-91.856***	-92.619***	-75.019***	-93.608***	-68.453***	-99.493***
	(6.093)	(3.183)	(1.915)	(2.808)	(4.089)	(5.556)	(2.297)	(2.707)
$\gamma : \mathbb{1}\{\geq 04-11\}$	10.504	4.793	16.310***	16.786***	22.067***	7.809	16.419***	1.031
	(7.757)	(3.441)	(2.403)	(3.509)	(5.314)	(6.615)	(2.995)	(2.998)
Adjusted R^2	0.154	0.142	0.129	0.131	0.127	0.128	0.139	0.128
N	35199	112774	290455	135802	71776	33360	144000	134970

***** 0.01 **0.05 * 0.1**

Note: The regressions include the zip-month fixed effects. The standard errors shown in the parenthesis are clustered at the zip code level.

In-home Digital

S73 Table: Effects of initial lockdown and stimulus payments on zip code level in-home digital spending

	Dollar Change ($)			Percentage Change (%)		
	All	Republican	Democratic	All	Republican	Democratic
$\beta : \mathbb{1}\{\geq 03-19\}$	28.612***	21.424***	36.567***	76.780***	69.672***	84.646***
	(0.659)	(0.710)	(1.132)	(2.053)	(2.836)	(2.973)
$\gamma : \mathbb{1}\{\geq 04-11\}$	49.302***	38.986***	60.322***	79.104***	85.013***	72.793***
	(1.046)	(1.049)	(1.833)	(2.877)	(3.960)	(4.184)
Adjusted R^2	0.382	0.315	0.403	0.147	0.143	0.152
N	854505	444974	409531	854505	444974	409531

***** 0.01 **0.05 * 0.1**

Note: The regressions include the zip-month fixed effects. The standard errors shown in the parenthesis are clustered at the zip code level.

S74 Table: Effects on in-home digital dollar change across geographic regions

	Dependent Variable: Dollar Change ($)							
	New England	Mideast	Southeast	Great Lakes	Plains	Rocky Mountain	Southwest	Far West
$\beta : \mathbb{1}\{\geq 03-19\}$	26.275***	21.482***	23.277***	32.575***	29.781***	30.683***	36.408***	34.417***
	(2.410)	(3.319)	(0.841)	(1.489)	(1.903)	(2.871)	(1.409)	(1.672)
$\gamma : \mathbb{1}\{\geq 04-11\}$	37.285***	71.779***	42.822***	65.295***	47.434***	42.116***	45.543***	28.731***
	(3.708)	(4.700)	(1.409)	(2.655)	(3.169)	(3.663)	(2.037)	(1.935)
Adjusted R^2	0.400	0.378	0.359	0.438	0.291	0.325	0.374	0.361
N	29998	118529	256379	130479	65832	27672	123420	102717

***** 0.01 **0.05 * 0.1**

Note: The regressions include the zip-month fixed effects. The standard errors shown in the parenthesis are clustered at the zip code level.

S75 Table: Effects on in-home digital percentage change across geographic regions

	Dependent Variable: Percentage Change (%)							
	New England	Mideast	Southeast	Great Lakes	Plains	Rocky Mountain	Southwest	Far West
$\beta : \mathbb{1}\{\geq 03-19\}$	79.535***	62.974***	68.457***	83.182***	75.710***	93.194***	81.972***	93.542***
	(11.481)	(6.377)	(3.596)	(5.274)	(7.180)	(11.237)	(4.616)	(6.458)
$\gamma : \mathbb{1}\{\geq 04-11\}$	60.509***	71.638***	84.366***	113.546***	103.484***	85.223***	72.744***	30.335***
	(15.697)	(7.890)	(5.148)	(7.522)	(10.289)	(14.994)	(7.311)	(8.208)
Adjusted R^2	0.166	0.195	0.131	0.152	0.136	0.130	0.129	0.132
N	29998	118529	256379	130479	65832	27672	123420	102717

***** 0.01 **0.05 * 0.1**

Note: The regressions include the zip-month fixed effects. The standard errors shown in the parenthesis are clustered at the zip code level.

In-home Non-digital

S76 Table: Effects of initial lockdown and stimulus payments on zip code level in-home non-digital spending

	Dollar Change ($)			Percentage Change (%)		
	All	Republican	Democratic	All	Republican	Democratic
$\beta : \mathbb{1}\{\geq 03-19\}$	-58.075***	-48.287***	-68.533***	-36.780***	-38.866***	-34.553***
	(1.286)	(1.413)	(2.182)	(1.277)	(1.835)	(1.770)
$\gamma : \mathbb{1}\{\geq 04-11\}$	192.403***	139.761***	246.650***	135.663***	130.363***	141.125***
	(2.831)	(2.582)	(4.989)	(1.783)	(2.486)	(2.557)
Adjusted R^2	0.302	0.218	0.335	0.119	0.122	0.116
N	1085064	557739	527325	1085064	557739	527325

***** 0.01 **0.05 * 0.1**

Note: The regressions include the zip-month fixed effects. The standard errors shown in the parenthesis are clustered at the zip code level.

S77 Table: Effects on in-home non-digital dollar change across geographic regions

	Dependent Variable: Dollar Change ($)							
	New England	Mideast	Southeast	Great Lakes	Plains	Rocky Mountain	Southwest	Far West
$\beta : \mathbb{1}\{\geq 03-19\}$	-39.551***	-65.282***	-73.296***	-63.783***	-42.230***	-18.750***	-54.163***	-39.213***
	(5.059)	(3.672)	(2.464)	(3.611)	(3.947)	(5.244)	(3.460)	(3.125)
$\gamma : \mathbb{1}\{\geq 04-11\}$	158.402***	242.202***	182.634***	260.140***	164.790***	115.148***	188.623***	132.781***
	(10.327)	(9.941)	(4.507)	(9.351)	(8.371)	(9.209)	(6.537)	(5.283)
Adjusted R^2	0.338	0.374	0.269	0.357	0.283	0.190	0.264	0.238
N	47585	151281	318850	159794	79531	35515	145829	147272

***** 0.01 **0.05 * 0.1**

Note: The regressions include the zip-month fixed effects. The standard errors shown in the parenthesis are clustered at the zip code level.

S78 Table: Effects on in-home non-digital percentage change across geographic regions

	Dependent Variable: Percentage Change (%)							
	New England	Mideast	Southeast	Great Lakes	Plains	Rocky Mountain	Southwest	Far West
$\beta : \mathbb{1}\{\geq 03-19\}$	-35.908***	-31.495***	-49.622***	-33.958***	-26.130***	-13.638*	-31.044***	-34.948***
	(6.559)	(3.520)	(2.373)	(3.500)	(4.881)	(7.272)	(2.930)	(3.407)
$\gamma : \mathbb{1}\{\geq 04-11\}$	152.252***	150.221***	133.485***	183.377***	139.433***	79.335***	116.547***	95.254***
	(8.803)	(4.608)	(3.165)	(5.146)	(6.846)	(9.250)	(4.614)	(4.436)
Adjusted R^2	0.138	0.133	0.108	0.133	0.117	0.095	0.113	0.104
N	47585	151281	318850	159794	79531	35515	145829	147272

***** 0.01 **0.05 * 0.1**

Note: The regressions include the zip-month fixed effects. The standard errors shown in the parenthesis are clustered at the zip code level.

.8 Other Non-food Shopping Spening

Total

S79 Table: Effects of initial lockdown and stimulus payments on zip code level other non-food shopping spending

	Dollar Change ($)			Percentage Change (%)		
	All	Republican	Democratic	All	Republican	Democratic
$\beta : \mathbb{1}\{\geq 03-19\}$	-173.994***	-115.334***	-275.578***	-23.030***	-20.193***	-27.944***
	(2.817)	(2.541)	(6.125)	(0.286)	(0.372)	(0.436)
$\gamma : \mathbb{1}\{\geq 04-11\}$	248.597***	187.981***	350.571***	27.530***	27.891***	26.922***
	(3.737)	(3.454)	(8.032)	(0.335)	(0.439)	(0.514)
Adjusted R^2	0.147	0.132	0.160	0.147	0.148	0.147
N	2174008	1371125	802883	2174008	1371125	802883

***** 0.01 **0.05 * 0.1**

Note: The regressions include the zip-month fixed effects. The standard errors shown in the parenthesis are clustered at the zip code level.

S80 Table: Effects on other non-food shopping dollar change across geographic regions

	Dependent Variable: Dollar Change ($)							
	New England	Mideast	Southeast	Great Lakes	Plains	Rocky Mountains	Southwest	Far West
$\beta : \mathbb{1}\{\geq 03-19\}$	-95.433***	-126.214***	-168.373***	-208.816***	-111.444***	-158.763***	-249.245***	-214.127***
	(7.168)	(5.801)	(5.035)	(8.200)	(6.350)	(12.740)	(10.677)	(8.872)
$\gamma : \mathbb{1}\{\geq 04-11\}$	122.444***	140.271***	305.031***	268.181***	193.032***	126.641***	389.154***	224.251***
	(8.426)	(6.183)	(7.550)	(10.679)	(11.436)	(14.196)	(13.632)	(9.796)
Adjusted R^2	0.116	0.115	0.141	0.142	0.123	0.144	0.170	0.175
N	108286	309646	655304	363466	209048	68541	243154	218766

***** 0.01 **0.05 * 0.1**

Note: The regressions include the zip-month fixed effects. The standard errors shown in the parenthesis are clustered at the zip code level.

S81 Table: Effects on other non-food shopping percentage change across geographic regions

	Dependent Variable: Percentage Change (%)							
	New England	Mideast	Southeast	Great Lakes	Plains	Rocky Mountains	Southwest	Far West
$\beta : \mathbb{1}\{\geq 03-19\}$	-28.115***	-26.893***	-20.355***	-27.974***	-17.858***	-23.164***	-16.789***	-27.437***
	(1.551)	(0.825)	(0.498)	(0.713)	(0.917)	(1.703)	(0.724)	(0.874)
$\gamma : \mathbb{1}\{\geq 04-11\}$	29.179***	25.053***	31.083***	29.073***	23.851***	19.129***	26.849***	23.723***
	(1.756)	(0.889)	(0.590)	(0.850)	(1.167)	(1.997)	(0.887)	(0.978)
Adjusted R^2	0.140	0.131	0.144	0.165	0.139	0.146	0.140	0.146
N	108286	309646	655304	363466	209048	68541	243154	218766

***** 0.01 **0.05 * 0.1**

Note: The regressions include the zip-month fixed effects. The standard errors shown in the parenthesis are clustered at the zip code level.

Personal Care and Cosmetics

S82 Table: Effects of initial lockdown and stimulus payments on zip code level personal care and cosmetics spending

	Dollar Change ($)			Percentage Change (%)		
	All	Republican	Democratic	All	Republican	Democratic
$\beta : \mathbb{1}\{\geq 03-19\}$	-14.319***	-5.902***	-20.029***	-12.938***	-10.506***	-14.588***
	(1.365)	(1.464)	(2.050)	(1.881)	(3.021)	(2.400)
$\gamma : \mathbb{1}\{\geq 04-11\}$	55.941***	37.198***	67.893***	75.386***	68.283***	79.916***
	(1.901)	(1.989)	(2.800)	(2.693)	(4.308)	(3.445)
Adjusted R^2	0.137	0.133	0.139	0.136	0.145	0.130
N	318170	126574	191596	318170	126574	191596

***** 0.01 **0.05 * 0.1**

Note: The regressions include the zip-month fixed effects. The standard errors shown in the parenthesis are clustered at the zip code level.

S83 Table: Effects on personal care and cosmetics dollar change across geographic regions

	Dependent Variable: Dollar Change ($)							
	New England	Mideast	Southeast	Great Lakes	Plains	Rocky Mountains	Southwest	Far West
$\beta : \mathbb{1}\{\geq 03-19\}$	-17.461***	-18.871***	-12.014***	-16.439***	5.390*	3.226	-27.281***	-4.889*
	(6.400)	(6.361)	(2.145)	(3.924)	(3.037)	(5.461)	(3.171)	(2.917)
$\gamma : \mathbb{1}\{\geq 04-11\}$	34.985***	62.190***	69.623***	61.987***	47.527***	31.805***	46.058***	31.449***
	(5.878)	(5.316)	(3.897)	(5.600)	(6.464)	(7.217)	(3.495)	(3.562)
Adjusted R^2	0.160	0.107	0.142	0.105	0.146	0.118	0.165	0.134
N	8204	36810	109023	41322	16827	8290	60816	36898

***** 0.01 **0.05 * 0.1**

Note: The regressions include the zip-month fixed effects. The standard errors shown in the parenthesis are clustered at the zip code level.

S84 Table: Effects on personal care and cosmetics percentage change across geographic regions

	Dependent Variable: Percentage Change (%)							
	New England	Mideast	Southeast	Great Lakes	Plains	Rocky Mountains	Southwest	Far West
$\beta : \mathbb{1}\{\geq 03-19\}$	-40.387***	-13.242**	-12.497***	-10.621*	5.132	8.752	-24.371***	-4.588
	(11.700)	(5.362)	(3.246)	(5.698)	(8.067)	(11.876)	(3.687)	(6.061)
$\gamma : \mathbb{1}\{\geq 04-11\}$	79.715***	79.779***	90.009***	80.200***	85.262***	55.695***	53.920***	53.684***
	(16.394)	(7.160)	(4.627)	(7.934)	(12.029)	(16.870)	(5.947)	(7.883)
Adjusted R^2	0.149	0.141	0.136	0.128	0.149	0.138	0.122	0.143
N	8204	36810	109023	41322	16827	8290	60816	36898

***** 0.01 **0.05 * 0.1**

Note: The regressions include the zip-month fixed effects. The standard errors shown in the parenthesis are clustered at the zip code level.

Auto Dealers

S85 Table: Effects of initial lockdown and stimulus payments on zip code level auto dealers spending

	Dollar Change ($)			Percentage Change (%)		
	All	Republican	Democratic	All	Republican	Democratic
$\beta : \mathbb{1}\{\geq 03-19\}$	-148.110***	-100.657***	-230.318***	-21.407***	-18.700***	-26.096***
	(2.473)	(2.273)	(5.350)	(0.278)	(0.363)	(0.421)
$\gamma : \mathbb{1}\{\geq 04-11\}$	176.803***	138.323***	241.582***	22.423***	23.153***	21.192***
	(2.830)	(2.747)	(5.952)	(0.321)	(0.422)	(0.486)
Adjusted R^2	0.146	0.132	0.157	0.153	0.151	0.156
N	2161833	1363697	798136	2161833	1363697	798136

***** 0.01 **0.05 * 0.1**

Note: The regressions include the zip-month fixed effects. The standard errors shown in the parenthesis are clustered at the zip code level.

S86 Table: Effects on auto dealers dollar change across geographic regions

	Dependent Variable: Dollar Change ($)							
	New England	Mideast	Southeast	Great Lakes	Plains	Rocky Mountains	Southwest	Far West
$\beta : \mathbb{1}\{\geq 03-19\}$	-75.202***	-98.484***	-150.578***	-169.396***	-98.843***	-130.759***	-217.269***	-180.625***
	(5.991)	(5.024)	(4.532)	(6.849)	(5.700)	(10.547)	(9.556)	(7.903)
$\gamma : \mathbb{1}\{\geq 04-11\}$	83.970***	100.126***	220.674***	193.932***	137.375***	83.286***	260.245***	161.170***
	(6.175)	(4.819)	(5.762)	(7.821)	(8.596)	(11.486)	(10.242)	(7.900)
Adjusted R^2	0.122	0.121	0.139	0.141	0.124	0.153	0.160	0.179
N	107072	306815	653282	361579	208003	68079	242159	217018

***** 0.01 **0.05 * 0.1**

Note: The regressions include the zip-month fixed effects. The standard errors shown in the parenthesis are clustered at the zip code level.

S87 Table: Effects on auto dealer percentage change across geographic regions

	Dependent Variable: Percentage Change (%)							
	New England	Mideast	Southeast	Great Lakes	Plains	Rocky Mountains	Southwest	Far West
$\beta : \mathbb{1}\{\geq 03-19\}$	-25.427***	-24.079***	-19.635***	-25.409***	-16.786***	-20.631***	-16.254***	-25.322***
	(1.477)	(0.812)	(0.481)	(0.697)	(0.901)	(1.646)	(0.711)	(0.850)
$\gamma : \mathbb{1}\{\geq 04-11\}$	23.311***	20.364***	25.447***	24.235***	19.252***	15.255***	21.178***	19.126***
	(1.681)	(0.847)	(0.566)	(0.806)	(1.105)	(1.938)	(0.866)	(0.941)
Adjusted R^2	0.143	0.140	0.148	0.169	0.145	0.150	0.143	0.152
N	107072	306815	653282	361579	208003	68079	242159	217018

***** 0.01 **0.05 * 0.1**

Note: The regressions include the zip-month fixed effects. The standard errors shown in the parenthesis are clustered at the zip code level.

Auto

S88 Table: Effects of initial lockdown and stimulus payments on zip code level auto spending

	Dollar Change ($)			Percentage Change (%)		
	All	Republican	Democratic	All	Republican	Democratic
$\beta : \mathbb{1}\{\geq 03-19\}$	-23.370***	-13.467***	-32.687***	-45.986***	-44.462***	-47.420***
	(2.249)	(2.508)	(3.660)	(3.532)	(5.139)	(4.861)
$\gamma : \mathbb{1}\{\geq 04-11\}$	78.466***	56.444***	97.968***	126.917***	126.602***	127.197***
	(2.650)	(3.020)	(4.172)	(5.029)	(7.635)	(6.648)
Adjusted R^2	0.088	0.076	0.093	0.107	0.113	0.100
N	501453	235157	266296	501453	235157	266296

***** 0.01 **0.05 * 0.1**

Note: The regressions include the zip-month fixed effects. The standard errors shown in the parenthesis are clustered at the zip code level.

S89 Table: Effects on auto dollar change across geographic regions

	Dependent Variable: Spending Change ($)							
	New England	Mideast	Southeast	Great Lakes	Plains	Rocky Mountains	Southwest	Far West
$\beta : \mathbb{1}\{\geq 03-19\}$	-24.084**	-19.202**	-20.513***	-52.417***	-12.042	-6.976	-27.953***	-4.106
	(10.312)	(8.289)	(3.294)	(8.529)	(9.075)	(8.235)	(5.097)	(6.214)
$\gamma : \mathbb{1}\{\geq 04-11\}$	80.684***	58.057***	67.664***	128.889***	89.262***	32.874***	87.335***	58.448***
	(11.401)	(8.058)	(3.873)	(9.414)	(11.154)	(9.470)	(6.757)	(6.639)
Adjusted R^2	0.087	0.073	0.063	0.067	0.067	0.059	0.166	0.090
N	14529	43998	168476	71525	32730	18626	94141	57516

***** 0.01 **0.05 * 0.1**

Note: The regressions include the zip-month fixed effects. The standard errors shown in the parenthesis are clustered at the zip code level.

S90 Table: Effects on auto percentage change across geographic regions

	Dependent Variable: Percentage Change (%)							
	New England	Mideast	Southeast	Great Lakes	Plains	Rocky Mountains	Southwest	Far West
$\beta : \mathbb{1}\{\geq 03-19\}$	-81.455***	-26.440**	-51.635***	-48.024***	-21.324	-30.355	-48.735***	-44.162***
	(23.746)	(13.156)	(5.747)	(10.797)	(13.347)	(18.429)	(7.219)	(11.641)
$\gamma : \mathbb{1}\{\geq 04-11\}$	189.207***	114.608***	102.755***	200.444***	165.923***	15.641	144.594***	89.341***
	(30.011)	(15.927)	(8.740)	(14.036)	(18.985)	(27.618)	(11.108)	(14.143)
Adjusted R^2	0.128	0.129	0.101	0.116	0.107	0.087	0.093	0.106
N	14529	43998	168476	71525	32730	18626	94141	57516

***** 0.01 **0.05 * 0.1**

Note: The regressions include the zip-month fixed effects. The standard errors shown in the parenthesis are clustered at the zip code level.

Used Goods and Pawn Stores

S91 Table: Effects of initial lockdown and stimulus payments on zip code level used goods and pawn stores spending

	Dollar Change ($)			Percentage Change (%)		
	All	Republican	Democratic	All	Republican	Democratic
$\beta : \mathbb{1}\{\geq 03-19\}$	10.111***	8.629***	11.581***	12.002***	12.023***	11.981***
	(1.745)	(2.040)	(2.825)	(2.763)	(3.944)	(3.871)
$\gamma : \mathbb{1}\{\geq 04-11\}$	101.536***	85.649***	115.573***	119.888***	116.071***	123.261***
	(3.155)	(3.906)	(4.792)	(4.660)	(6.890)	(6.323)
Adjusted R^2	0.116	0.107	0.122	0.131	0.139	0.124
N	378128	184384	193744	378128	184384	193744

***** 0.01 **0.05 * 0.1**

Note: The regressions include the zip-month fixed effects. The standard errors shown in the parenthesis are clustered at the zip code level.

S92 Table: Effects on used goods and pawn stores dollar change across geographic regions

	Dependent Variable: Dollar Change ($)							
	New England	Mideast	Southeast	Great Lakes	Plains	Rocky Mountains	Southwest	Far West
$\beta : \mathbb{1}\{\geq 03-19\}$	5.012	-10.007**	15.136***	0.277	11.877**	0.181	15.932***	7.041**
	(7.275)	(3.986)	(3.068)	(3.981)	(4.721)	(6.910)	(4.780)	(3.427)
$\gamma : \mathbb{1}\{\geq 04-11\}$	38.291***	31.367***	107.089***	80.481***	89.638***	63.913***	152.035***	72.301***
	(10.770)	(5.467)	(5.500)	(7.577)	(9.876)	(10.321)	(7.869)	(9.044)
Adjusted R^2	0.160	0.125	0.098	0.098	0.094	0.083	0.160	0.126
N	9039	28459	119000	57899	30302	17544	70205	45805

***** 0.01 **0.05 * 0.1**

Note: The regressions include the zip-month fixed effects. The standard errors shown in the parenthesis are clustered at the zip code level.

S93 Table: Effects on used goods and pawn stores percentage change across geographic regions

	Dependent Variable: Percentage Change (%)							
	New England	Mideast	Southeast	Great Lakes	Plains	Rocky Mountains	Southwest	Far West
$\beta : \mathbb{1}\{\geq 03-19\}$	-6.555	-21.937*	21.189***	-4.934	9.695	-6.340	22.948***	8.660
	(20.589)	(11.663)	(4.831)	(7.743)	(9.957)	(11.934)	(5.482)	(8.781)
$\gamma : \mathbb{1}\{\geq 04-11\}$	66.530*	101.891***	124.723***	101.032***	116.050***	70.446***	154.232***	92.030***
	(34.838)	(16.723)	(8.093)	(12.585)	(16.481)	(17.929)	(9.969)	(15.343)
Adjusted R^2	0.178	0.154	0.129	0.131	0.127	0.101	0.124	0.135
N	9039	28459	119000	57899	30302	17544	70205	45805

***** 0.01 **0.05 * 0.1**

Note: The regressions include the zip-month fixed effects. The standard errors shown in the parenthesis are clustered at the zip code level.

.9 Finance Spending

Total

S94 Table: Effects of initial lockdown and stimulus payments on zip code level finance spending

	Dollar Change ($)			Percentage Change (%)		
	All	Republican	Democratic	All	Republican	Democratic
$\beta : \mathbb{1}\{\geq 03-19\}$	-775.464***	-489.050***	-1163.787***	-38.025***	-35.613***	-41.296***
	(11.316)	(9.700)	(22.356)	(0.653)	(0.931)	(0.880)
$\gamma : \mathbb{1}\{\geq 04-11\}$	923.597***	727.005***	1184.250***	62.169***	68.161***	54.224***
	(14.358)	(12.820)	(28.497)	(0.855)	(1.224)	(1.138)
Adjusted R^2	0.377	0.323	0.395	0.141	0.135	0.146
N	1670577	958054	712523	1670577	958054	712523

***** 0.01 **0.05 * 0.1**

Note: The regressions include the zip-month fixed effects. The standard errors shown in the parenthesis are clustered at the zip code level.

S95 Table: Effects on finance dollar change across geographic regions

	Dependent Variable: Spending Change ($)							
	New England	Mideast	Southeast	Great Lakes	Plains	Rocky Mountains	Southwest	Far West
$\beta : \mathbb{1}\{\geq 03-19\}$	-453.322***	-829.654***	-733.128***	-778.259***	-542.099***	-485.186***	-1031.536***	-908.187***
	(31.485)	(31.526)	(20.610)	(30.314)	(29.191)	(36.143)	(36.673)	(33.358)
$\gamma : \mathbb{1}\{\geq 04-11\}$	546.890***	556.139***	1182.689***	969.229***	627.359***	542.260***	1296.828***	791.638***
	(36.001)	(25.078)	(32.488)	(39.842)	(36.734)	(42.889)	(41.960)	(33.209)
Adjusted R^2	0.328	0.381	0.352	0.515	0.346	0.320	0.303	0.307
N	80507	247810	516314	250674	131882	49594	202204	192967

***** 0.01 **0.05 * 0.1**

Note: The regressions include the zip-month fixed effects. The standard errors shown in the parenthesis are clustered at the zip code level.

S96 Table: Effects on finance percentage change across geographic regions

	Dependent Variable: Percentage Change (%)							
	New England	Mideast	Southeast	Great Lakes	Plains	Rocky Mountains	Southwest	Far West
$\beta : \mathbb{1}\{\geq 03-19\}$	-37.512***	-41.497***	-35.680***	-40.459***	-37.292***	-36.089***	-30.165***	-47.015***
	(3.216)	(1.816)	(1.161)	(1.895)	(2.457)	(3.788)	(1.424)	(1.777)
$\gamma : \mathbb{1}\{\geq 04-11\}$	64.407***	45.915***	74.033***	76.831***	51.289***	45.418***	57.151***	49.754***
	(4.201)	(2.032)	(1.512)	(2.515)	(3.266)	(5.679)	(2.194)	(2.174)
Adjusted R^2	0.132	0.138	0.135	0.146	0.128	0.121	0.144	0.130
N	80507	247810	516314	250674	131882	49594	202204	192967

***** 0.01 **0.05 * 0.1**

Note: The regressions include the zip-month fixed effects. The standard errors shown in the parenthesis are clustered at the zip code level.

Financial Services

S97 Table: Effects of initial lockdown and stimulus payments on zip code level financial services spending

	Dollar Change ($)			Percentage Change (%)		
	All	Republican	Democratic	All	Republican	Democratic
$\beta : \mathbb{1}\{\geq 03-19\}$	-760.931***	-479.203***	-1137.023***	-37.312***	-35.092***	-40.276***
	(11.265)	(9.686)	(22.095)	(0.635)	(0.902)	(0.867)
$\gamma : \mathbb{1}\{\geq 04-11\}$	904.882***	711.014***	1157.895***	61.277***	67.159***	53.600***
	(14.256)	(12.757)	(28.093)	(0.834)	(1.189)	(1.126)
Adjusted R^2	0.381	0.328	0.398	0.141	0.135	0.144
N	1627000	926965	700035	1627000	926965	700035

***** 0.01 **0.05 * 0.1**

Note: The regressions include the zip-month fixed effects. The standard errors shown in the parenthesis are clustered at the zip code level.

S98 Table: Effects on financial services dollar change across geographic regions

	Dependent Variable: Spending Change ($)							
	New England	Mideast	Southeast	Great Lakes	Plains	Rocky Mountains	Southwest	Far West
$\beta : \mathbb{1}\{\geq 03-19\}$	-446.758***	-821.922***	-712.364***	-772.772***	-537.574***	-463.808***	-1010.266***	-883.648***
	(31.730)	(31.347)	(20.463)	(30.321)	(29.489)	(34.903)	(36.403)	(32.922)
$\gamma : \mathbb{1}\{\geq 04-11\}$	531.746***	536.492***	1164.560***	953.741***	614.599***	517.216***	1255.006***	775.897***
	(35.858)	(24.944)	(32.122)	(39.809)	(36.752)	(41.999)	(41.255)	(33.015)
Adjusted R^2	0.337	0.395	0.350	0.521	0.352	0.330	0.307	0.318
N	77474	241118	505430	241644	127273	47872	198656	188826

***** 0.01 **0.05 * 0.1**

Note: The regressions include the zip-month fixed effects. The standard errors shown in the parenthesis are clustered at the zip code level.

S99 Table: Effects on financial services percentage change across geographic regions

	Dependent Variable: Percentage Change (%)							
	New England	Mideast	Southeast	Great Lakes	Plains	Rocky Mountains	Southwest	Far West
$\beta : \mathbb{1}\{\geq 03-19\}$	-34.894***	-40.654***	-34.895***	-41.868***	-36.721***	-32.020***	-30.442***	-44.345***
	(3.147)	(1.775)	(1.121)	(1.818)	(2.424)	(3.820)	(1.406)	(1.742)
$\gamma : \mathbb{1}\{\geq 04-11\}$	59.616***	42.265***	74.049***	78.529***	50.334***	48.250***	55.681***	48.200***
	(4.023)	(1.979)	(1.480)	(2.440)	(3.154)	(5.573)	(2.105)	(2.175)
Adjusted R^2	0.133	0.137	0.134	0.149	0.126	0.115	0.144	0.127
N	77474	241118	505430	241644	127273	47872	198656	188826

***** 0.01 **0.05 * 0.1**

Note: The regressions include the zip-month fixed effects. The standard errors shown in the parenthesis are clustered at the zip code level.

Charity

S100 Table: Effects of initial lockdown and stimulus payments on zip code level charity spending

	Dollar Change ($)			Percentage Change (%)		
	All	Republican	Democratic	All	Republican	Democratic
$\beta : \mathbb{1}\{\geq 03-19\}$	-15.932***	-15.180***	-16.445***	-37.775***	-39.640***	-36.501***
	(1.490)	(2.208)	(2.003)	(3.661)	(5.838)	(4.698)
$\gamma : \mathbb{1}\{\geq 04-11\}$	21.109***	16.918***	23.476***	49.644***	49.715***	49.604***
	(1.948)	(2.612)	(2.664)	(5.234)	(8.557)	(6.612)
Adjusted R^2	0.143	0.143	0.143	0.149	0.165	0.139
N	202561	83010	119551	202561	83010	119551

***** 0.01 **0.05 * 0.1**

Note: The regressions include the zip-month fixed effects. The standard errors shown in the parenthesis are clustered at the zip code level.

S101 Table: Effects on charity dollar change across geographic regions

	Dependent Variable: Dollar Change ($)							
	New England	Mideast	Southeast	Great Lakes	Plains	Rocky Mountains	Southwest	Far West
$\beta : \mathbb{1}\{\geq 03-19\}$	-4.785	-26.210***	-14.618***	-13.318***	-21.238***	-33.208***	-19.114***	-6.511*
	(7.425)	(5.411)	(2.460)	(4.036)	(6.202)	(9.866)	(3.615)	(3.767)
$\gamma : \mathbb{1}\{\geq 04-11\}$	5.888	18.249**	23.700***	23.735***	18.974**	24.368***	27.407***	11.187***
	(8.448)	(7.116)	(3.340)	(5.270)	(8.830)	(8.074)	(4.835)	(4.240)
Adjusted R^2	0.166	0.121	0.156	0.137	0.137	0.142	0.136	0.146
N	6381	19345	66175	27274	11413	5887	36211	29923

***** 0.01 **0.05 * 0.1**

Note: The regressions include the zip-month fixed effects. The standard errors shown in the parenthesis are clustered at the zip code level.

S102 Table: Effects on charity percentage change across geographic regions

	Dependent Variable: Percentage Change (%)							
	New England	Mideast	Southeast	Great Lakes	Plains	Rocky Mountains	Southwest	Far West
$\beta : \mathbb{1}\{\geq 03-19\}$	-4.542	-52.481***	-39.951***	-42.724***	-27.252**	-72.490***	-35.511***	-23.691**
	(23.807)	(12.215)	(6.139)	(10.599)	(12.944)	(20.872)	(8.555)	(10.203)
$\gamma : \mathbb{1}\{\geq 04-11\}$	-7.266	33.046**	62.350***	48.877***	61.986***	58.870**	61.291***	27.746**
	(31.535)	(14.857)	(9.126)	(14.749)	(23.076)	(29.501)	(12.973)	(13.349)
Adjusted R^2	0.182	0.164	0.144	0.153	0.169	0.163	0.131	0.153
N	6381	19345	66175	27274	11413	5887	36211	29923

***** 0.01 **0.05 * 0.1**

Note: The regressions include the zip-month fixed effects. The standard errors shown in the parenthesis are clustered at the zip code level.

.10 Personal Services

S103 Table: Effects of initial lockdown and stimulus payments on zip code level personal services spending

	Dollar Change ($)			Percentage Change (%)		
	All	Republican	Democratic	All	Republican	Democratic
$\beta : \mathbb{1}\{\geq 03-19\}$	-130.514***	-90.951***	-165.939***	-73.247***	-67.626***	-78.279***
	(2.116)	(2.194)	(3.407)	(0.933)	(1.463)	(1.185)
$\gamma : \mathbb{1}\{\geq 04-11\}$	44.593***	30.275***	56.104***	27.074***	29.766***	24.910***
	(1.697)	(2.030)	(2.572)	(1.113)	(1.809)	(1.383)
Adjusted R^2	0.137	0.108	0.151	0.125	0.129	0.118
N	856842	403201	453641	856842	403201	453641

***** 0.01 **0.05 * 0.1**

Note: The regressions include the zip-month fixed effects. The standard errors shown in the parenthesis are clustered at the zip code level.

S104 Table: Effects on personal services dollar change across geographic regions

	Dependent Variable: Dollar Change ($)							
	New England	Mideast	Southeast	Great Lakes	Plains	Rocky Mountains	Southwest	Far West
$\beta : \mathbb{1}\{\geq 03-19\}$	-80.938***	-124.113***	-131.467***	-144.310***	-85.389***	-112.084***	-176.433***	-106.272***
	(6.338)	(6.117)	(3.834)	(6.323)	(5.778)	(8.041)	(6.547)	(4.154)
$\gamma : \mathbb{1}\{\geq 04-11\}$	32.023***	50.791***	53.588***	66.949***	32.537***	-0.350	28.864***	31.706***
	(6.040)	(5.263)	(2.941)	(5.411)	(5.859)	(7.692)	(4.796)	(3.531)
Adjusted R^2	0.116	0.122	0.126	0.104	0.125	0.120	0.203	0.105
N	30800	106773	268091	118643	57212	28243	127676	119789

***** 0.01 **0.05 * 0.1**

Note: The regressions include the zip-month fixed effects. The standard errors shown in the parenthesis are clustered at the zip code level.

S105 Table: Effects on personal services percentage change across geographic regions

	Dependent Variable: Percentage Change (%)							
	New England	Mideast	Southeast	Great Lakes	Plains	Rocky Mountains	Southwest	Far West
$\beta : \mathbb{1}\{\geq 03-19\}$	-69.170***	-77.579***	-68.128***	-80.476***	-69.926***	-80.875***	-67.957***	-81.194***
	(5.308)	(2.704)	(1.651)	(2.610)	(3.701)	(5.340)	(2.033)	(2.692)
$\gamma : \mathbb{1}\{\geq 04-11\}$	14.920**	23.741***	31.024***	39.737***	25.567***	12.299*	20.174***	23.223***
	(6.108)	(3.056)	(1.884)	(3.184)	(4.962)	(7.156)	(2.634)	(2.997)
Adjusted R^2	0.133	0.134	0.122	0.125	0.121	0.122	0.133	0.106
N	30800	106773	268091	118643	57212	28243	127676	119789

***** 0.01 **0.05 * 0.1**

Note: The regressions include the zip-month fixed effects. The standard errors shown in the parenthesis are clustered at the zip code level.

.11 Clothing and Accessories

S106 Table: Clothing and accessories spending under COVID-19 pandemic

	Spending Change ($)			Percentage Change (%)		
	All	Republican	Democratic	All	Republican	Democratic
$\beta : \mathbb{1}\{\geq 03-19\}$	-324.618***	-177.429***	-471.633***	-56.691***	-53.564***	-59.813***
	(6.157)	(4.127)	(11.146)	(0.674)	(1.035)	(0.863)
$\gamma : \mathbb{1}\{\geq 04-11\}$	261.294***	157.653***	360.322***	68.147***	72.282***	64.195***
	(5.472)	(3.674)	(9.869)	(0.913)	(1.390)	(1.190)
Adjusted R^2	0.186	0.132	0.205	0.138	0.136	0.137
N	1015916	505099	510817	1015916	505099	510817

***** 0.01 **0.05 * 0.1**

Note: The regressions use zip-month fixed effects. Standard errors shown in the parenthesis are clustered at zip-code level.

S107 Table: Clothing and accessories spending difference by geographic regions

	Dependent Variable: Spending Change ($)							
	New England	Mideast	Southeast	Great Lakes	Plains	Rocky Mountain	Southwest	Far West
$\beta : \mathbb{1}\{\geq 03-19\}$	-252.130***	-461.892***	-304.582***	-445.548***	-247.786***	-138.111***	-327.233***	-201.671***
	(21.278)	(24.917)	(9.010)	(23.317)	(19.276)	(12.274)	(12.278)	(9.375)
$\gamma : \mathbb{1}\{\geq 04-11\}$	185.896***	403.238***	254.768***	423.481***	221.734***	78.357***	142.248***	133.981***
	(15.832)	(22.043)	(7.226)	(20.968)	(16.358)	(10.827)	(7.464)	(7.040)
Adjusted R^2	0.178	0.225	0.160	0.159	0.129	0.120	0.271	0.119
N	39656	138634	332507	141176	68878	29859	139292	126296

***** 0.01 **0.05 * 0.1**

Note: The regressions use zip-month fixed effects. Standard errors shown in the parenthesis are clustered at zip-code level.

S108 Table: Clothing and accessories percentage difference by geographic regions

	Dependent Variable: Percentage Change (%)							
	New England	Mideast	Southeast	Great Lakes	Plains	Rocky Mountains	Southwest	Far West
$\beta : \mathbb{1}\{\geq 03-19\}$	-58.810***	-57.114***	-57.628***	-60.383***	-55.963***	-49.851***	-53.467***	-54.562***
	(4.041)	(1.875)	(1.156)	(1.856)	(2.584)	(4.246)	(1.585)	(2.024)
$\gamma : \mathbb{1}\{\geq 04-11\}$	65.343***	69.791***	74.623***	92.707***	72.804***	49.417***	43.398***	49.318***
	(4.533)	(2.209)	(1.568)	(2.647)	(3.516)	(6.016)	(2.203)	(2.629)
Adjusted R^2	0.156	0.150	0.133	0.142	0.143	0.108	0.145	0.112
N	39656	138634	332507	141176	68878	29859	139292	126296

***** 0.01 **0.05 * 0.1**

Note: The regressions use zip-month fixed effects. Standard errors shown in the parenthesis are clustered at zip-code level.
